# Differential skewing of donor-unrestricted and **γδ** T cell repertoires in tuberculosis-infected human lungs

**DOI:** 10.1172/JCI130711

**Published:** 2019-11-25

**Authors:** Paul Ogongo, Adrie J.C. Steyn, Farina Karim, Kaylesh J. Dullabh, Ismael Awala, Rajhmun Madansein, Alasdair Leslie, Samuel M. Behar

**Affiliations:** 1 Africa Health Research Institute and; 2 School of Laboratory Medicine, Nelson Mandela School of Medicine, University of KwaZulu-Natal, Durban, South Africa.; 3 Institute of Primate Research, National Museums of Kenya, Nairobi, Kenya.; 4 Department of Cardiothoracic Surgery, Nelson Mandela School of Medicine, University of KwaZulu-Natal, Durban, South Africa.; 5 Department of Infection and Immunity, University College London, London, United Kingdom.; 6 Department of Microbiology and Physiological Systems, University of Massachusetts Medical School, Worcester, Massachusetts, USA.

**Keywords:** Immunology, Infectious disease, Adaptive immunity, T-cell receptor, Tuberculosis

## Abstract

Unconventional T cells that recognize mycobacterial antigens are of great interest as potential vaccine targets against tuberculosis (TB). This includes donor-unrestricted T cells (DURTs), such as mucosa-associated invariant T cells (MAITs), CD1-restricted T cells, and γδ T cells. We exploited the distinctive nature of DURTs and γδ T cell receptors (TCRs) to investigate the involvement of these T cells during TB in the human lung by global TCR sequencing. Making use of surgical lung resections, we investigated the distribution, frequency, and characteristics of TCRs in lung tissue and matched blood from individuals infected with TB. Despite depletion of MAITs and certain CD1-restricted T cells from the blood, we found that the DURT repertoire was well preserved in the lungs, irrespective of disease status or HIV coinfection. The TCRδ repertoire, in contrast, was highly skewed in the lungs, where it was dominated by Vδ1 and distinguished by highly localized clonal expansions, consistent with the nonrecirculating lung-resident γδ T cell population. These data show that repertoire sequencing is a powerful tool for tracking T cell subsets during disease.

## Graphical abstract

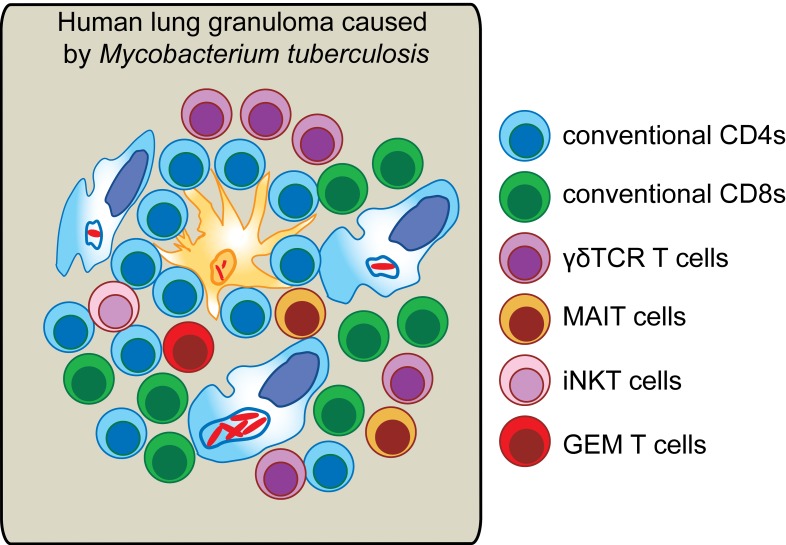

## Introduction

Tuberculosis (TB), caused by *Mycobacterium tuberculosis* (Mtb), remains the leading cause of death from an infectious agent (Global Tuberculosis Report, WHO, 2018 (1). Although treatable with antibiotics, there is an urgent need to develop an effective vaccine against TB because of the challenges of diagnosis, the long duration of treatment, and the rise of drug-resistant strains. Protection from disease in 90% of infected individuals demonstrates that immune responses can cope with Mtb infection (2). Bacille Calmette-Guérin (BCG), the current vaccine, protects infants from disseminated TB and may enhance immunity if readministered, or when given by intravenous or aerosol vaccination routes. In addition, BCG can be improved upon, as shown by the recent phase IIb trial of the novel M72/AS01E vaccine (3). These data offer hope that an improved TB vaccine is possible, but more potent candidates are needed.

The potential to harness donor-unrestricted T cells (DURTs) and other unconventional T cells to boost anti-TB immunity is of great interest to the vaccine field (4). Conventional T cells are “restricted” to recognizing peptide antigens bound to MHC molecules that are highly polymorphic between unrelated individuals. Unconventional T cells, in contrast, generally recognize antigens bound to nonpolymorphic antigen-presenting molecules and are thus “unrestricted” by the host genotype (5). In addition, they typically target conserved pathogen-derived lipids and metabolites, which are less likely to mutate and be lost as immune targets.

DURTs described to date include mucosa-associated invariant T cells (MAITs), HLA-E–restricted T cells, invariant NK T cells (iNKTs), and group 1 CD1–restricted T cells including germline-encoded mycolyl lipid–reactive T cells (GEMs). MAITs, iNKTs, and GEMs all recognize their cognate antigens (bacterial metabolites bound to MR-1 or lipid-derived ligands bound to CD1a, -b, -c, or -d) via αβ T cell receptors (TCRs). In addition, γδ T cells are a major class of unconventional T cells that recognize a variety of peptide and nonpeptide antigens presented by CD1 or other nonpolymorphic molecules via the γδ TCR (6, 7)

Many studies indicate that these unconventional T cells play an important protective role in TB, particularly during early infection (8–10). For example, γδ T cells recognize Mtb antigens, respond to BCG vaccination, suppress mycobacterial growth, and confer protection when adoptively transferred, and expansion of pulmonary γδ T cells by vaccination reduces disease pathology in nonhuman primates (NHPs) (11). Likewise, CD1-restricted DURTs recognize mycobacterial lipids, transfer of mycolic acid–specific CD1b-restricted T cells confers protection against TB to humanized mice, and airway LAM-responsive, CD1b-restricted T cells are associated with protection from disease in TB-exposed humans (12–14). MR1-KO mice, which lack MAITs, show a reduced ability to control initial infection (15), and polymorphism associated with reduced MR1 expression in humans is linked to TB susceptibility and meningeal disease (16). This anti-TB activity of DURTs and γδ T cells and the universal nature of their presenting molecules make the highly conserved antigens they recognize attractive vaccine targets (9, 16, 17). Another promising feature of DURTs and some γδ subsets is their apparent preference to migrate to and reside at mucosal sites. Promotion of lung residency of TB-specific T cells is thought to be essential for the protective activity of these cells, and this protection may even be highly localized within the particular tissue, as T cell control can vary among different lesions within the same lung (18). This may also explain why it is challenging to identify strong T cell correlates of protection in the blood (19–22). MAITs, for example, are highly enriched at mucosal barriers, including in the lung, where they comprise 2%–4% of T cells (8). Several infections, including TB, are associated with a loss of MAITs from the circulation, which could result from recruitment to infected tissues (8). Likewise, pulmonary CD1–restricted αβ T cells and γδ T cells isolated from Mtb-infected subjects rapidly migrate back to the lung after intravenous infusion (13, 23). However, little is known about the lung DURT and γδ T cell response in human TB infection, as most studies have focused on blood. The existence of noncirculating tissue-resident memory T cells (Trms) (24) demonstrates that T cell responses in the blood and tissue do not always mirror each other. Therefore, it is necessary to characterize DURT and γδ T cell responses in the lung during active TB infection to understand the potential contribution of nonclassical T cell subsets to immunity.

Unlike conventional T cells, DURTs use TCRs that are less diverse and frequently shared between individuals, which may result from the nonpolymorphic nature of the presenting molecules and the relatively restricted nature of their antigens. This is particularly true for TCRα chains of DURTs, which are frequently formed by canonical rearrangements of Vα (TRAV) and Jα (TRAJ) gene segments (5). For example, MAITs exclusively use TRAV1-02 paired with TRAJ33 (25, 26), and iNKTs use an invariant TCRα chain formed by TRAV10 joining to TRAJ18 (Table 1 and see Supplemental material; supplemental material available online with this article; https://doi.org/10.1172/JCI130711DS1). Importantly, both subsets are expanded in all humans, even at a very young age (5). Likewise, GEMs use TRAV1-02 and are widely detected. Indeed, a strategy to discover new DURTs involves the identification of expanded and highly shared TCRα chains through TCR sequencing (27). Finally, although γδ TCR diversity is similar to that of αβ T cells, it is largely achieved through extensive junctional diversity, as γδ TCRs have a reduced number of V and J gene segments, with an overwhelming bias toward Vγ9 paired with Vδ2 (7).

In this study, we exploited the distinctive and highly shared nature of DURTs and γδ TCRs to determine the involvement of these unconventional T cells in the immune response to TB infection using a global TCR-sequencing approach. We studied a cohort of HIV-infected and uninfected subjects undergoing surgical lung resection to treat ongoing TB or the sequela of treated infection and determined the distribution, frequency, and characteristics of DURT and γδ TCR clonotypes in blood and matched lung tissue from subjects with active TB or previous TB disease. In addition, we assessed the impact of HIV coinfection on the DURT and γδ TCR repertoire.

## Results

### Patient cohort and their TCR repertoire.

**Figure 1:**
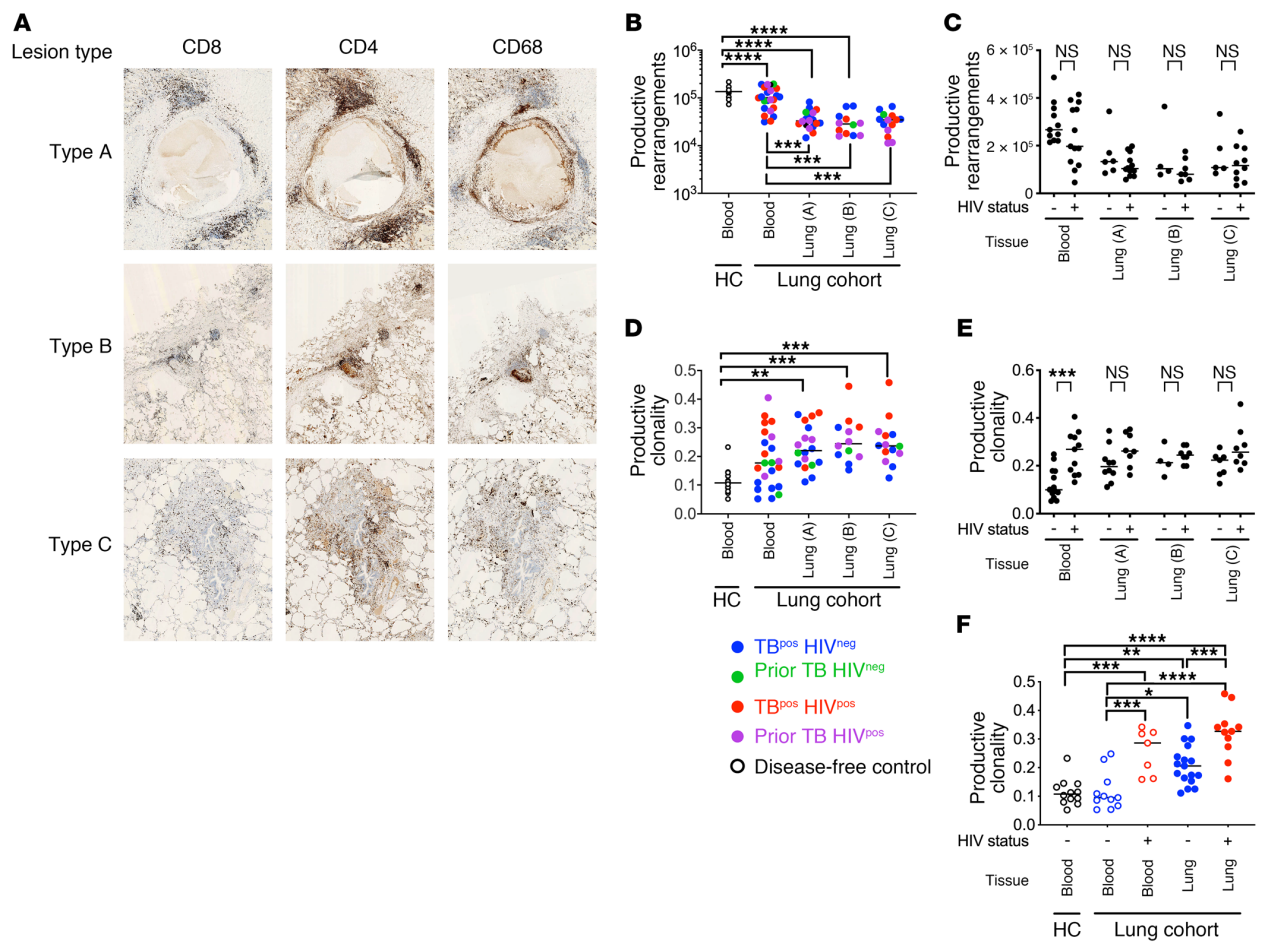
TCR sequences from human TB granulomas. (**A**) Representative immunohistology images of lung tissue from an HIV^neg^ subject with active TB (original magnification, ×600) showing CD4^+ve^ and CD8^+ve^ T cells and CD68^+ve^ macrophages. Samples from type A tissue, the most diseased area of the resected lung, had classic caseous granulomas with a distinct lymphocyte cuff and lymphocyte aggregates. Samples from types B and C, from less diseased tissue, show lymphocytic infiltrations and uninvolved alveoli. (**B**) Number of unique productive TCRα and TCRδ rearrangements obtained from deep-level (blood) or survey-level (lung) sequencing. Each point represents a unique subject. Color identifies the clinical status, grouped by tissue and lung lesion type (i.e., type A, B, or C). (**C**) Number of unique productive rearrangements obtained from blood or lung tissue from HIV^neg^ versus seropositive subjects (HIV^pos^) with active or prior TB. (**D**) Clonality of the productive TCRα and TCRδ rearrangements (as in **B**). (**E**) Productive clonality calculated for TCRs identified in blood or lung from HIV^neg^ versus HIV^pos^ subjects with active or prior TB. (**F**) Effect of HIV status on TCR clonality during active TB. Error bars indicate the median. *P < 0.05, **P < 0.01, ***P < 0.001, and ****P < 0.0001, by Kruskal-Wallis 1-way ANOVA with Dunn’s multiple comparisons test (**B**–**F**).

To study immunity at the site of disease, we recruited 31 subjects undergoing partial lung resection for clinical indications. HIV-coinfected (HIV^pos^) or HIV-seronegative (HIV^neg^) subjects were classified as having active TB or prior TB on the basis of clinical findings (Table 1). Individual lung tissue blocks from severely diseased (type A), moderately affected (type B), or less-severely affected (type C) lung areas were categorized by the operating surgeon and informed by preoperative imaging studies. Representative lung immunohistology showed lymphocytic infiltrates consisting of CD4, CD8, and CD68 staining and necrosis (particularly in type A lesions) (Figure 1A). The considerable variability between individuals prevented the categorization of subjects on the basis of lung pathology (e.g., cavitary disease, calcification). Importantly, we detected extensive lung involvement in all lung samples including type C lesions.

We determined the TCRα/δ rearrangements by high-throughput sequencing of 81 samples of blood, lung, and sputum from the 31 subjects described and of blood from 12 healthy (disease-free) controls (HCs) (28, 29). TCR sequences corresponding to more than 29 million templates, which encoded 224,000 unique TCRα/δ aa sequences, were analyzed, and more than half were productive rearrangements (lung: 60% ± 2%; PBMCs: 57% ± 2%). Deep sequencing of PBMC samples acquired 280,000 ± 114,000 productive TCRα/δ sequences, whereas survey-level sequencing of lung samples obtained 124,000 ± 75,000. Blood from HCs contained more unique productive rearrangements than did blood from the lung cohort (132,000 ± 36,000 vs. 116,000 ± 60,000) (Figure 1B). Fewer unique TCRα/δ sequences were identified in lung (33,000 ± 17,000) than in blood, in part because survey-level sequencing was used in the lung. HIV status had no significant effect on the number of productive rearrangements (Figure 1C).

We examined TCR clonality, which represents the tendency of a T cell population to be dominated by clonally expanded T cells and can signify antigen-driven responses (Supplemental material). We found that the lung TCR repertoire was significantly more clonal than the HC blood TCR repertoire (Figure 1D). HIV status affected blood TCR clonality, which was quite variable among lung cohort subjects (Figure 1E). For HIV^neg^ subjects, TCR clonality was significantly higher in lung than in blood (Figure 1F). HIV infection was associated with higher clonality in the blood and lungs of HIV^pos^ subjects compared with HIV^neg^ subjects, which might reflect expansion of the HIV-specific T cell and/or contraction of the TCR repertoire through CD4 depletion. These findings support the idea that the lung is enriched with clonally expanded T cells compared with PBMCs because of recruitment and expansion of antigen-specific T cells at the site of disease (29).

### Frequency of DURTs and γδ T cells during active TB.

**Figure 2:**
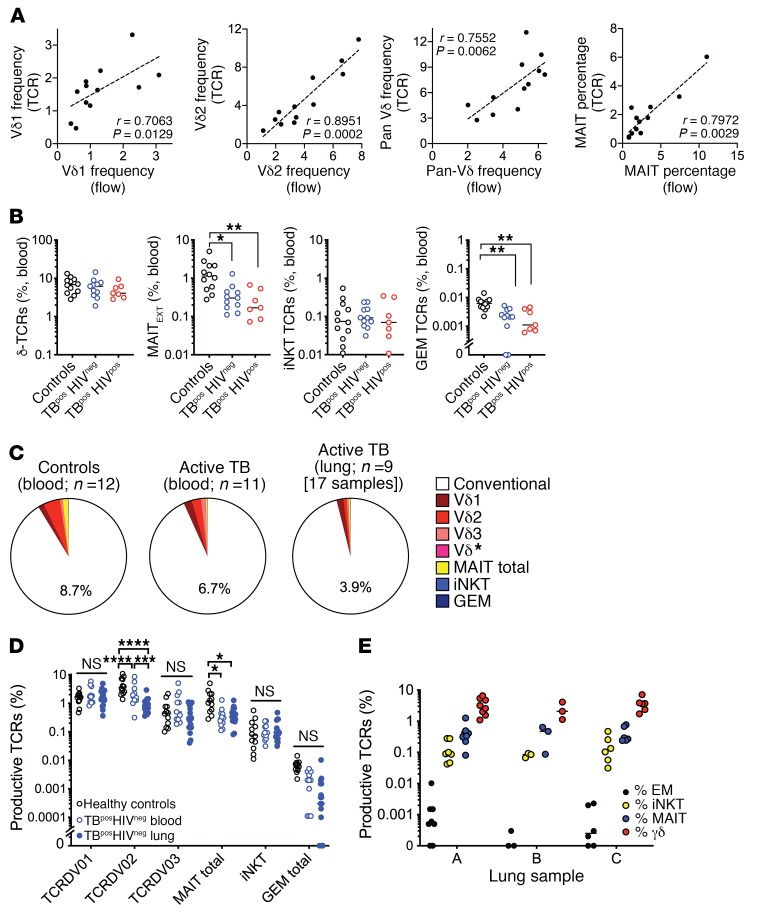
Next-generation sequencing summary for the human TCRA/D locus. (**A**) Frequency of Vδ1, Vδ2, γδ T cells and MAITs as determined by TCR sequencing (*y* axis) or flow cytometry (*x* axis, fraction of CD3^+^ T cells) in blood from HCs. (**B**) Percentage of γδ T cells, MAITs, iNKTs, and GEMs in blood from HCs, HIV^pos^ subjects, and HIV^neg^ subjects with active TB. (**C**) Percentage of DURTs in blood from HCs (*n* = 12) and in blood (*n* = 11) or lung tissue (*n* = 17) from HIV^pos^ subjects with active pulmonary TB. The TCRδ sequences encoded by the Vδ1, Vδ2, or Vδ3 or Vδ* (Vδ4+Vδ5+Vδ6+Vδ7+Vδ8) are identified by different shades of red. The non-DURTs are defined as “conventional.” (**D**) Percentage of Vδ1, Vδ2, or Vδ3, MAIT, iNKT, and GEM T cells in blood from HCs and blood and lung tissue from HIV^neg^ subjects with active pulmonary TB. (**E**) Percentage of total GEM, MAITs, iNKT, and γδ T cells in lung lesion types A, B, and C. *P < 0.05, **P < 0.01, ***P < 0.001, and ****P < 0.0001, by Kruskal-Wallis 1-way ANOVA with Dunn’s multiple comparisons test (**B**) and 2-way ANOVA with Tukey’s multiple comparisons test (**D** and **E**).

To determine the frequency of GEMs, MAITs, iNKTs, and γδ T cells, we identified TCRα/δ features representing different T cell subsets (Table 2). First, we confirmed the quantitative nature of our approach by analyzing the TCR repertoire of HCs using sequencing and flow cytometry. γδ T cells were identified with a pan-γδ TCR antibody and subdivided on the basis of Vδ1 or Vδ2. We classified MAITs according to expression of Vα7.2 (TRAV1-02), CD161, and CD26 (16). The frequency of γδ and MAIT TCRs in the same samples was based on TCR characteristics (Table 2). We found a strong correlation between the TCR frequencies, as determined by flow cytometry and sequencing (Figure 2A). Thus, high-throughput TCR analysis represents a valid method for enumerating DURT subsets.

We next determined whether the frequency of unconventional T cells varied between HCs and subjects with active TB. Blood from HCs had a reproducible hierarchy, with the frequency of γδ T cells > MAITs > iNKTs > GEMs (Figure 2B). Although this hierarchy was preserved during active TB, the frequencies of GEMs and MAITs were significantly lower in the blood of subjects with active TB, independent of HIV status (Figure 2B).

To determine whether DURT TCRs were enriched in infected lung tissue compared with blood, we analyzed lung TCRs from HIV^neg^ subjects with active TB, as HIV can affect the frequency of some DURTs (30–33). We found that αβ DURTs and γδ TCRs accounted for nearly 9% of TCR sequences in the blood of HCs, which was slightly higher than for HIV^neg^ subjects with active TB (Figure 2C). The frequency of αβ DURTs and γδ TCRs was even lower (~4%) in the 17 lung lesions from 9 HIV^neg^ subjects with active TB, arguing against a general enrichment of DURTs in the lung. These differences were driven by reductions in the abundance of Vδ2 TCRs. The frequency TCRDV2, which is the dominant Vδ gene used by γδ T cells in the blood, was 2.7-fold lower in the lungs of HIV^neg^ subjects with active TB compared with matched blood samples or blood from HCs (*P* < 0.0001) (Figure 2D). The frequency of MAIT TCRs in the blood and lungs of subjects with active TB was also significantly lower than in the blood of HCs. In contrast, the frequency of iNKTs was similar in all 3 groups. The frequency of GEM TCRs was not significantly different between groups, although these TCRs were present at a very low frequency in the lungs and were undetectable in some subjects. Finally, distribution of the 4 T cell subsets was similar in the 3 types of lung lesions examined (Figure 2E), indicating that there was no association between DURT frequency and disease severity in our cohort. Thus, all 4 types of DURT subsets were identified in the lungs of subjects with active TB. Next, we explored differences in the TCR repertoires of these different DURT subsets.

### CD1-restricted T cells in the lungs.

**Figure 3:**
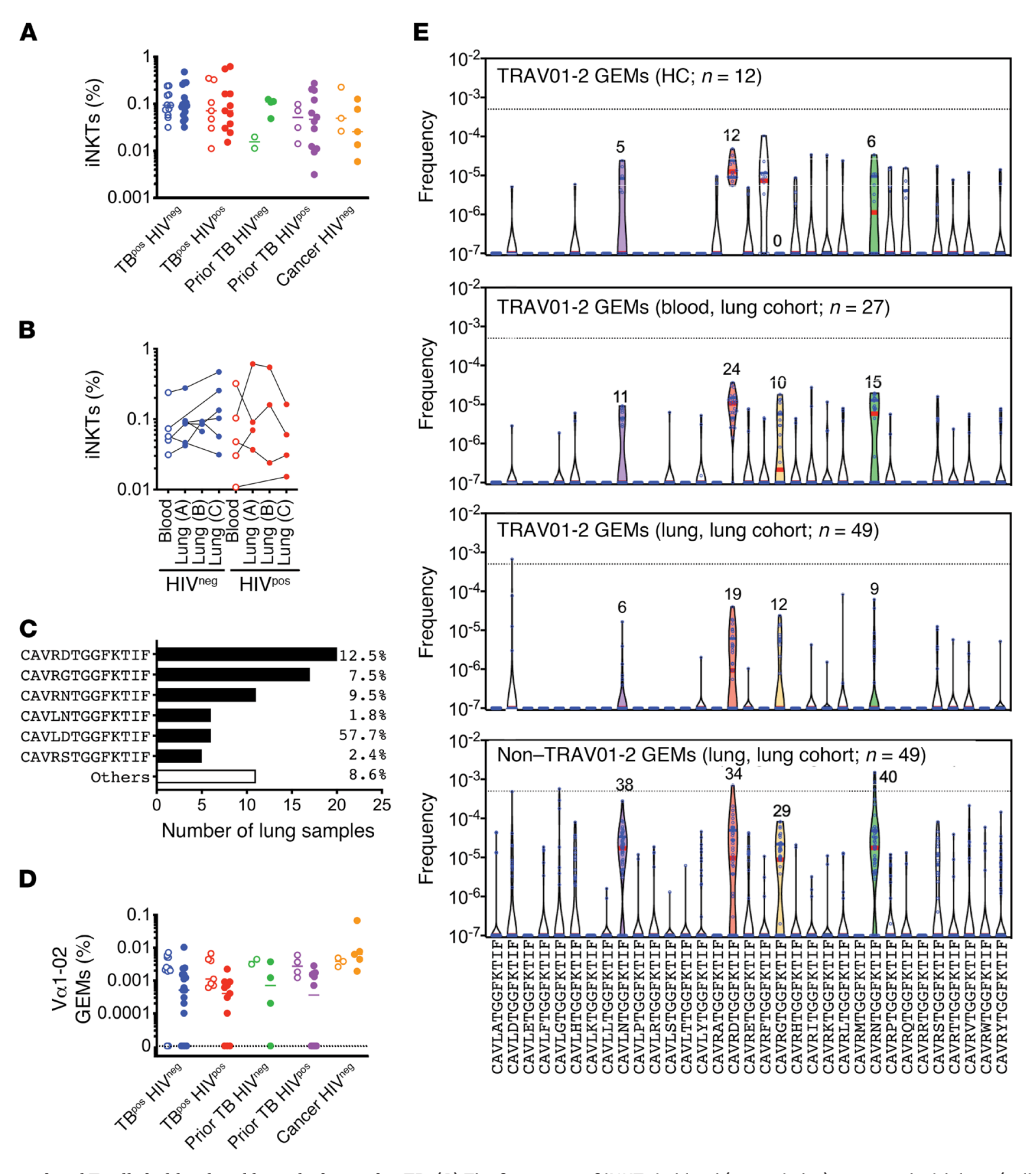
CD1-restricted T cells in blood and lung during active TB. (**A**) The frequency of iNKTs in blood (open circles) compared with lung (solid circles) among 5 clinical populations. (**B**) iNKT cell frequency in paired lung and blood samples, stratified by HIV status for subjects with active TB. Each line represents a single individual. (**C**) Using a consensus definition of GEM TCRα (Vα1-02, Jα9, CDR3α=CAV(R/L)xTGGFKTIF), GEMs were identified in lung tissue. The 6 most frequently detected clonotypes are shown, with their total relative abundance in the aggregated lung TCR data set. (**D**) Frequency of GEMs in blood (open circles) versus lung tissue (solid circles) among 5 clinical populations. (**E**) Clonotypes encoded by TRAV01-2 with the CDR3α CAV(R/L)xTGGFKTIF were identified in HCs (blood, top) and subjects in the lung cohort (blood, second graph; lung, third graph). Non–TRAV01-2 clonotypes encoding the CAV(R/L)xTGGFKTIF CDR3α were identified in lung (bottom graph). A frequency of 0.0000001 was assigned to clonotypes not detected. Dotted lines indicate a productive frequency of 0.05%. Colored violins indicate the most frequently detected lung clonotypes. Red lines indicate the median and blue lines the quartiles.

Of the known CD1-restricted T cells, iNKTs are the best described. The Vα10/Jα18 recombination that creates the invariant CDR3α of iNKTs (CVVSDRGSTLGRLYF) was detected in 78 of 79 samples (Figure 3A). The frequency of this iNKT TCRα was highly variable and ranged from 0.003% to 0.5% of the lung TCR repertoire. However, the iNKT TCRα was not significantly enriched in the lungs compared with the blood, nor did its frequency differ between HIV^neg^ and HIV^pos^ subjects (Figure 3A). Recognizing the significant individual-to-individual variation, we analyzed the distribution of iNKTs between paired blood and lung samples from subjects with active TB (Figure 3B) and found no discernable pattern. Thus, although iNKTs were present in the blood and lungs of nearly all subjects, we did not detect an increase in pulmonary iNKTs in any of the groups.

In addition to CD1d-restricted T cells (i.e., NKTs), T cells that are restricted to group 1 (CD1a, -b, and -c) recognize lipid and glycolipids from the cell wall of Mtb (5), although only a handful with validated CD1 restriction and known antigen specificity have been sequenced (34, 35). Of these, we searched for 5 CDR3α sequences and identified 3, which were associated with the Jα originally described to be used by the lipid-reactive T cell clones (Supplemental Figure 1). These sequences used a diverse Vα gene and were present at low frequencies.

CD1b-restricted T cells that recognize glucose monomycolate (GMM) share TCRα genes and CDR3α motifs (5, 36, 37). The CD1b-restricted, GMM-specific T cell clone 18 uses TRAV01-02 and TRAJ09, with the CDR3α sequence CAVRNTGGFKTIF (37). This rearrangement is typical of GMM-specific CD1b-restricted T cells (i.e., GEMs) and may represent a semi-invariant rearrangement (37). This possibility is strengthened by the detection of the CAV[R/L]xTGGFKTIF CDR3α motif among PBMCs from Mtb-infected subjects and among T cells enriched using GMM-CD1b tetramers (38). Among the TRAV01-02/TRAJ09 rearrangements with this motif, the most frequently detected CDR3α sequences in lung were CAVRDTGGFKTIF (*n* = 19 of 49), CAVRGTGGFKTIF (*n* = 12 of 49), and CAVRNTGGFKTIF (*n* = 9 of 49) (Figure 3C). Although CAVRDTGGFKTIF was the most frequently detected, CAVLDTGGFKTIF had the greatest aggregate frequency because it was expanded in subject 09-213. Although GEM TCRs were frequently detected within our cohort, their abundance was generally low, and they did not differ between disease groups (Figure 3D).

**Figure 4:**
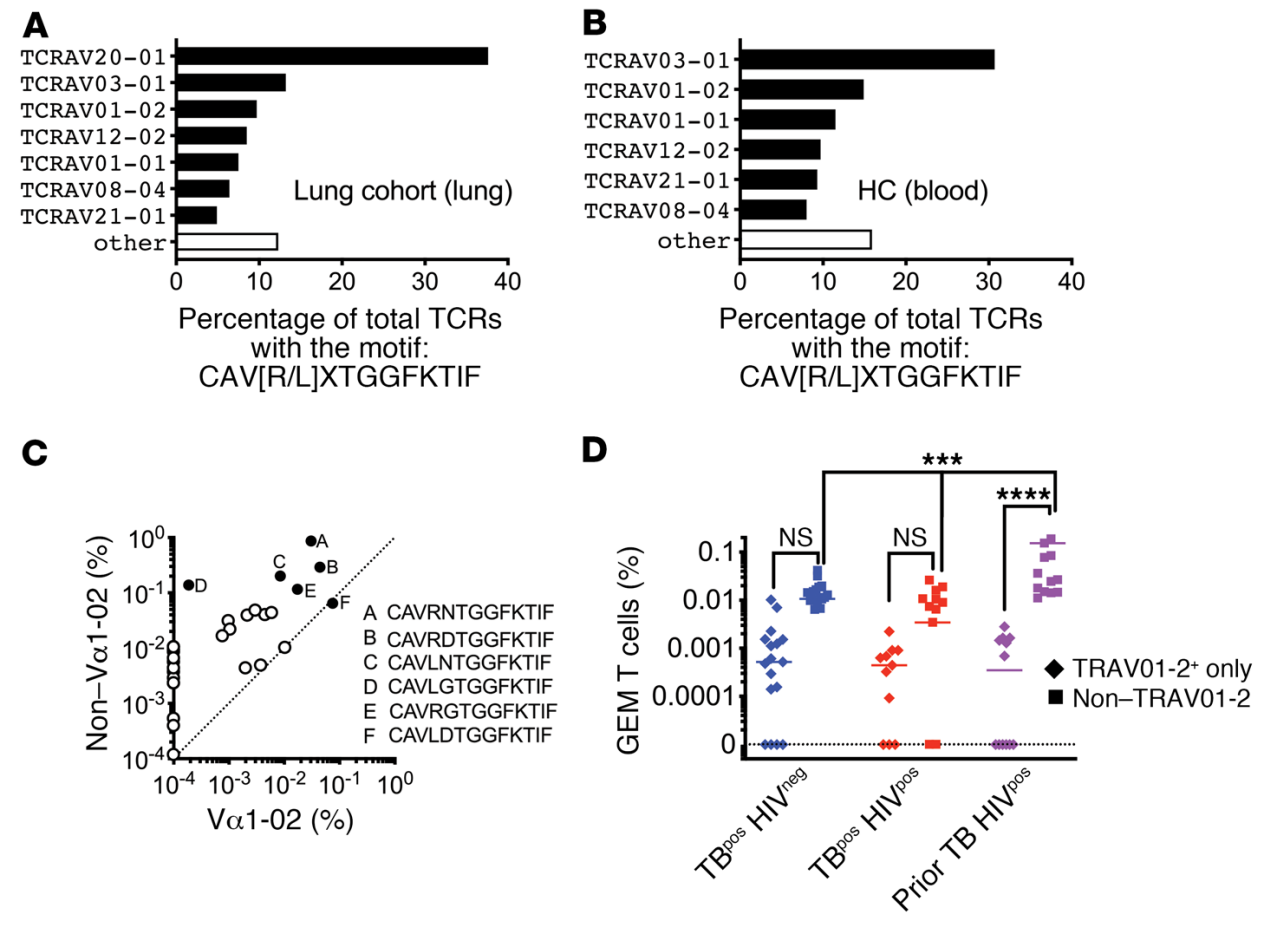
CD1-restricted T cells in blood and lung during active TB. (**A**) TRAV diversity among TCRs from the lung cohort (blood, lung, sputum) with a consensus sequence of CAV(R/L)xTGGFKTIF. (**B**) TRAV diversity among TCRs from HC blood with a consensus sequence of CAV(R/L)xTGGFKTIF. (**C**) Relative abundance of CAV(R/L)xTGGFKTIF among TRAV01-2 and non–TRAV01-2 cell populations. Each circle represents a unique clonotype (solid circles represent the 6 most abundant clonotypes). (**D**) Frequency of GEM and GEM-like TCRs in lung tissue from 3 clinical populations. Some statistical comparisons have been omitted for clarity. ***P < 0.001 and ****P < 0.0001, by ordinary 2-way ANOVA with Tukey’s multiple comparisons test. Error bars indicate the median.

In addition to the TRAV01-02^+^ GEM-like T cells, we also identified non–TRAV01-02 clonotypes with a CDR3α sequence of CAV[R/L]xTGGFKTIF, all of which used the TRAJ09 gene. These GEM-like TCRα sequences were detected in the blood of all subjects (*n* = 39 of 39) and in most lung samples (*n* = 47 of 49), consistent with the highly shared nature of other non–MHC-restricted TCRs (Figure 3E and Supplemental Figure 2). Similar to TRAV01-02 clonotypes, CAVRDTGGFKTIF, CAVLNTGGFKTIF, CAVRNTGGFKTIF, and CAVRGTGGFKTIF were frequently detected and abundant. We analyzed the lung cohort samples and found that the GEM-like TCRs most commonly used TRAV03-1 instead of TRAV01-02 (TRAV20 was predominantly used by a single subject) (Figure 4, A and B). These data led us to consider the relative abundance of CAV[R/L]xTGGFKTIF sequences among TRAV01-02 versus non–TRAV01-02 TCRs. These sequences were more frequent among the non–TRAV01-02 TCRs, and certain CDR3α sequences were only found among the non–TRAV01-02 TCRs (Figure 4C). The frequency of lung T cells with the CAV[R/L]xTGGFKTIF motif was greater among the non–TRAV01-02 TCRs than the TRAV01-02 TCRs, sometimes by more than 100-fold in the 3 largest patient groups from the lung cohort (Figure 4D). Finally, the frequency of GEM-like TCRs was significantly increased in the lungs of HIV^pos^ subjects with prior TB compared with those with active TB (Figure 4D). These data suggest that HIV coinfection, per se, does not deplete GEM-like TCRs in the lung, as occurs in the blood. Thus, these data establish that CD1-restricted T cells are present in the lungs during human TB infection. We propose that the non–TRAV01-02 TCRs that encode the CAV[R/L]xTGGFKTIF motif may represent a large population of GMM-specific CD1-restricted T cells.

### MAITs in the lung.

**Figure 5:**
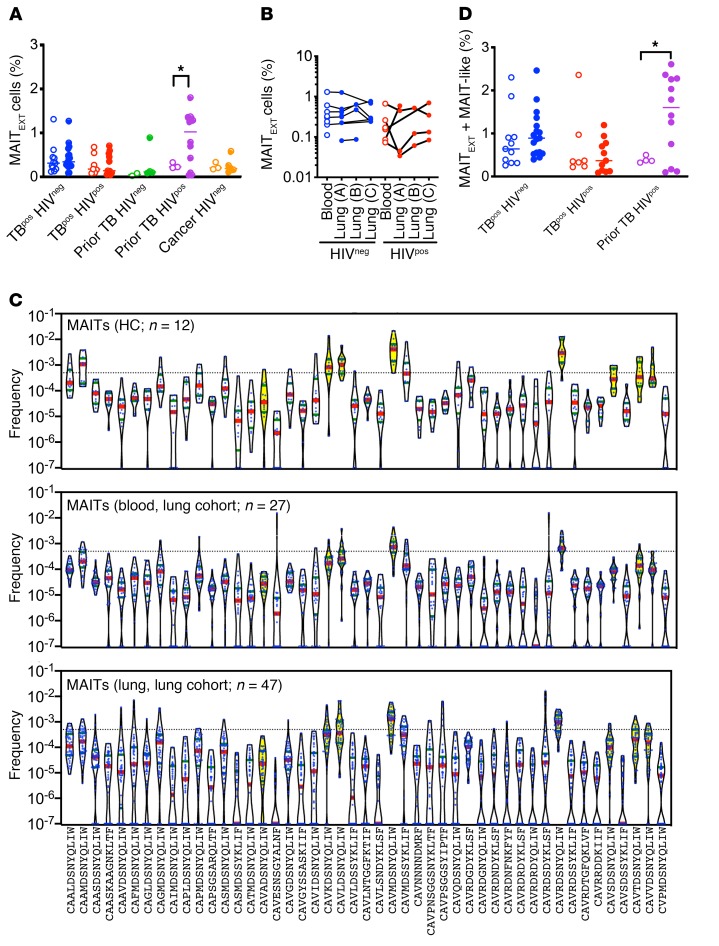
MAITs are expanded in the lungs of HIV^pos^ subjects with a history of TB. (**A**) Frequency of extended MAITs (MAIT_Ext_, Table 2) in blood (open circles) and lung (solid circles) among 5 clinical populations. (**B**) Frequency of MAIT_Ext_ in paired lung and blood from subjects with active TB, stratified by HIV status. Each line represents a single individual. (**C**) MAIT_Ext_ and MAIT-like clonotypes in HC blood (top graph) and in samples form the lung cohort (blood, middle graph; lung, bottom graph). For clonotypes not detected, a frequency of 0.0000001 was assigned. Dotted line indicates a productive frequency of 0.05%. MAIT_Ext_ clonotypes are designated by violins in yellow. Red bars in indicate the median; blue bars indicate quartiles. (**D**) Frequency of total MAIT TCRs (MAIT_Ext_ + MAIT-like) in blood (open circles) and lung (solid circles) among 5 clinical populations. *P **<** 0.05, by 2-way ANOVA with Sidak’s multiple comparisons test (**A** and **D**). Error bars indicate the median.

MAITs are semi-invariant T cells defined by their TCRα chain, which is a recombination of Vα1-02 with Jα33, resulting in the CDR3α sequence CAVxDSNYQLIW and referred to hereafter as MAIT_EXT_ (Table 2) (26). The frequency of MAITs in blood, as measured by flow cytometry, is reduced during TB, with or without HIV coinfection (30, 31, 39). We observed a reduction in MAIT TCR frequency during active TB, without any reciprocal enrichment in the lungs (Figure 2, B and D). We detected a trend toward lower MAIT cell frequencies in subjects with HIV coinfection, however, it was not statistically significant. Moreover, we found that MAITs were significantly elevated in the lungs of HIV^pos^ subjects with prior TB, suggesting that, as with GEM-like T cells, the loss of MAITs from lung tissue is not a consistent feature of HIV infection (Figure 5A). As with iNKTs, we did not observe marked differences in the frequency of MAITs between paired blood and lung samples (Figure 5, A and B).

Since the antigens that MR1-restricted MAITs recognize are still being defined, the definition of what constitutes a MAIT TCR is still in flux (16). We extended our definition of MAITs to include TCRs that scored 1.0 on the MAIT match, although not all of these have been confirmed to be MR1 restricted. Given this expanded definition of MAIT-like cells, we used 47 CDR3α sequences to query our data (Table 2). Importantly, not all of these were encoded by Vα1-02 or Jα33. We determined the frequencies of each MAIT_EXT_ or MAIT-like clonotype for each study subject (Figure 5C). We detected 7 of 8 MAIT_EXT_ clonotypes in the blood of all 12 HCs, and 4 were found in all blood and lung samples from the lung cohort (Figure 5C). Over half of the MAIT-like clonotypes were also detected in 100% of the HC blood samples, but at a lower frequency than the MAIT_EXT_ clonotypes (*P* < 0.0001, Mann-Whitney *U* test). In general, the MAIT-like clonotypes were highly shared among subjects in the lung cohort, which is consistent with the behavior of these clonotypes as DURTs (Figure 5C). However, we did not observe enrichment of MAIT-like sequences in subjects with TB infection compared with HCs. Finally, we lowered the MAIT match score to 0.950–0.999 and identified 600 CDR3α regions among our samples that resembled MAIT TCRs. Analysis of these TCRs did not reveal any private expansions in subjects with active TB, although, again, we did find some expansions in HIV^pos^ subjects with prior TB (Supplemental Figure 3).

Given that MAIT_EXT_ TCRα chains were more frequently detected in the lungs of HIV^pos^ subjects with prior TB, we asked whether MAIT-like TCR clonotypes contributed to the lung MAIT repertoire in subjects with TB with or without HIV coinfection. We calculated the frequency of total MAITs (e.g., MAIT_EXT_ plus MAIT-like). As before, we found that HIV^pos^ subjects with a history of prior TB had a greater frequency of total MAIT TCRs in their lung lesions compared with their blood (Figure 5D). This supports the conclusion that, unlike in blood, HIV infection does not deplete lung MAITs.

### Vδ1/Jδ1 TCRs are prominent in the lungs of HIV^neg^ subjects with TB.

**Figure 6:**
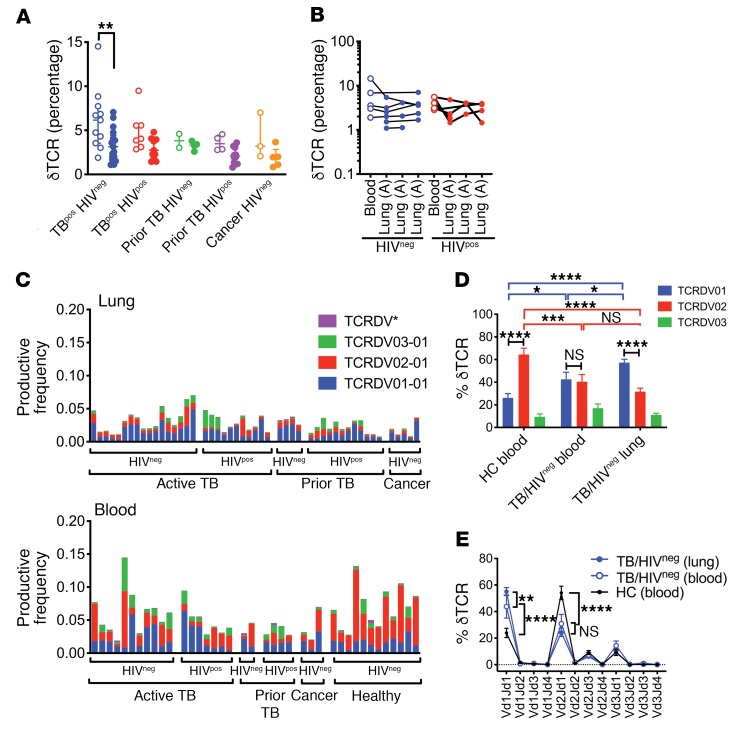
Distribution of unique TCRδ clonotypes. (**A**) Frequency of γδ T cells (Table 2) in blood (open circles) and lung (solid circles) among 5 clinical populations. (**B**) Frequency of γδ T cells in paired lung and blood samples from subjects with active TB were stratified by HIV status. Each line represents a single individual. (**C**) Vδ1, Vδ2, Vδ3, or Vδ* (Vδ4+Vδ5+Vδ6+Vδ7+Vδ8) use for each lung (top graph) or blood (bottom graph) sample. The clinical status of each subject is shown. (**D**) Vδ1, Vδ2, or Vδ3 use by T cells from the blood of HCs versus blood or lung tissue of HIV^neg^ subjects with active pulmonary TB. Some statistical comparisons have been omitted for clarity. (**E**) Pairing analysis of Vδ1, Vδ2, or Vδ3 with Jδ1, Jδ2, Jδ3, or Jδ4 in blood from HCs compared with blood and lung tissue from HIV^neg^ subjects with active pulmonary TB. *P < 0.05, **P < 0.01, ***P < 0.001, and ****P < 0.0001, by 2-way ANOVA with Sidak’s (**A**) or Tukey’s (**E**) multiple comparisons test. Error bars indicate the median; data represent the mean ± SEM.

The frequency of γδ T cells (defined in Table 2 and Supplemental Figure 4) was lower in the lungs than in the blood only for HIV^neg^ subjects with active TB (Figure 6A) but did not vary between lung samples (Figure 6B). We quantified Vδ gene use and found an overall reduction of Vδ2 in lung compared with blood (Figure 6, C and D). Although Vδ2 was clearly dominant in HCs, Vδ2 and Vδ1 were codominant in the blood of subjects with active TB (Figure 6E). The dominant Vδ gene in the lung was Vδ1, and this difference was highly significant in HIV^neg^ subjects with active TB, who had an increased frequency of Vδ1/Jδ1 rearrangements and a reciprocal reduction in the relative amount of Vδ2/Jδ1 (Figure 6, C–E). These data suggest that the γδ T cell repertoire is highly skewed in lungs compared with blood.

### Identification of enriched and abundant TCRδ clonotypes in the lung.

**Figure 7:**
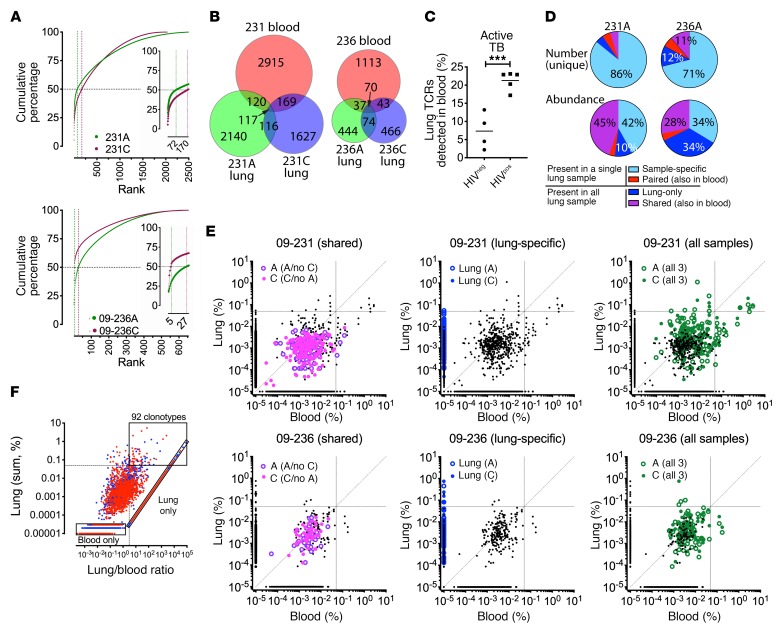
TCRδ clonotypes detected in TB lung granulomas. (**A**) Cumulative frequency of lung TCRδ clonotypes from 2 representative HIV^neg^ subjects with active TB (09-231 and 09-236). Vertical dotted lines indicate the number of clonotypes comprising 50% of the total lung TCRδs. (**B**) Overlap of TCRδ clonotypes detected in the blood and lung tissue of subjects 09-231 and 09-236. (**C**) Percentage of lung TCRδ clonotypes detected in the blood (i.e., common to blood and lung) for subjects with active TB. (**D**) Number of TCRδ clonotypes unique to lung samples from subjects 09-231A and 09-236A, shared with blood only, shared with other lung samples, or detected in all 3 samples (top pie charts). Relative abundance of the TCRδ clonotypes in these groups (bottom pie charts). (**E**) Groups of TCRδ clonotypes differed in their abundance. Lung frequency of each TCRδ clonotype versus its blood frequency. For clonotypes not detected, the frequency was assigned 0.00000011. All unique clonotypes are shown in black. Specific groups of clonotypes are shown in color as follows: paired (shared between 1 lung sample and blood; purple); lung-specific (blue); or shared (in all 3 samples; green). Open circles indicate the frequency in lung type A lesions; solid circles indicate the frequency in lung type B lesions. Horizontal and vertical lines equal 0.05%. Diagonal is the line of equivalency. (**F**) Identification of abundant and lung-enriched TCRδ clonotypes. For each of 32,000 unique clonotypes from 9 subjects, the sum of the frequencies in lung versus the lung/PBMC ratio for each clonotype was plotted. For clonotypes not detected, the frequency was assigned 0.00000011. Abundant and enriched clonotypes were defined as having a sum frequency of greater than 0.05% (horizontal dotted line) and a lung/PBMC ratio of greater than 3 (vertical dotted line). Blue represents HIV^neg^; red represents HIV^pos^. Bottom left and diagonal boxes indicate clonotypes detected only in blood or lung, and top right box indicates 92 abundant and enriched clonotypes. ***P < 0.001, by Student’s *t* test. Error bars indicate the median.

Having identified 32,000 unique TCRδ clonotypes, we next identified TCRδs that were overrepresented in the infected lung. Figure 7A highlights 2 representative HIV^neg^ individuals with active TB (subjects 09-231 and 09-236). For lung samples from subjects 09-231A and 09-231C, 2.9% and 8.4% of the unique TCRδ clonotypes accounted for 50% of the total TCRδ sequences. Similarly, 4.3% and 0.6% of the unique TCRδs in lung samples from subjects 09-236A and 09-236C accounted for 50% of the total TCRδ sequences.

As the lung samples were surgical specimens, contamination with blood lymphocytes was unavoidable. We next assessed each TCRδ clonotype by comparing matched blood and lung samples. Remarkably, only 7% of the TCRδ clonotypes identified in the lung tissue were detected in blood, despite the fact that we used a greater sequencing depth for blood (Figure 7, B and C). The overlap was significantly greater for HIV^pos^ individuals than for HIV^neg^ individuals. Different lung samples taken from the same individual had largely nonoverlapping γδ TCR repertoires, indicating considerable heterogeneity within the lung tissue (Figure 7B).

We classified lung TCRδs into 4 groups: (a) unique to a single lung sample (sample-specific); (b) present in both lung samples but not in the blood (lung only); (c) shared between 1 lung sample and blood (paired); or (d) present in all lung samples and blood (shared) (Figure 7D). Most TCRδs were detected in a single lung sample. As many of these clonotypes were present at a low frequency, they could represent blood contamination. In contrast, those unique clonotypes found in both lung samples were abundant and overrepresented (Figure 7D). For example, for subjects 09-231 and 09-236, 9.4% and 23% of unique TCRs were present in both lung samples; together, they accounted for 55% and 62%, respectively, of the total γδ TCRs (Figure 7D).

To formally identify overrepresented TCRδ clonotypes in the lung, we plotted the sum-total frequency for each clonotype in lung versus blood for TCRδs that were paired, lung-only, or shared (Figure 7E). The majority of paired clonotypes were present at a low frequency and generally more abundant in the blood than in the lung. This pattern is consistent with TCRδs that are present in the lungs as a result of blood contamination (Figure 7E, left). The clonotypes that were detected only in the lung tissue had a higher overall frequency (Figure 7E, middle). Finally, the clonotypes that were detected in all samples contained TCRs that were enriched either in lung or blood (Figure 7E, right). To identify the TCRδ clonotypes that were both abundant and preferentially expressed in the lungs, we analyzed each of the 9 subjects with active TB separately, pooled the data, and identified 92 clonotypes with a productive frequency of greater than 0.05% and a lung/blood ratio of greater than 3 (Figure 7F). We found strong evidence of highly expanded γδ T cells within TB-infected tissue that were either absent, or present at a very low frequency, in the blood. In contrast to the other DURT TCRs, this is consistent with a significant lung-resident, nonrecirculating γδ T cell population in the lungs. Importantly, many of TCRδs that were expanded in the lung would have been missed by studies that focused on γδ T cell responses in the blood.

### Profiling of enriched and abundant TCRδ clonotypes in the lung.

**Figure 8:**
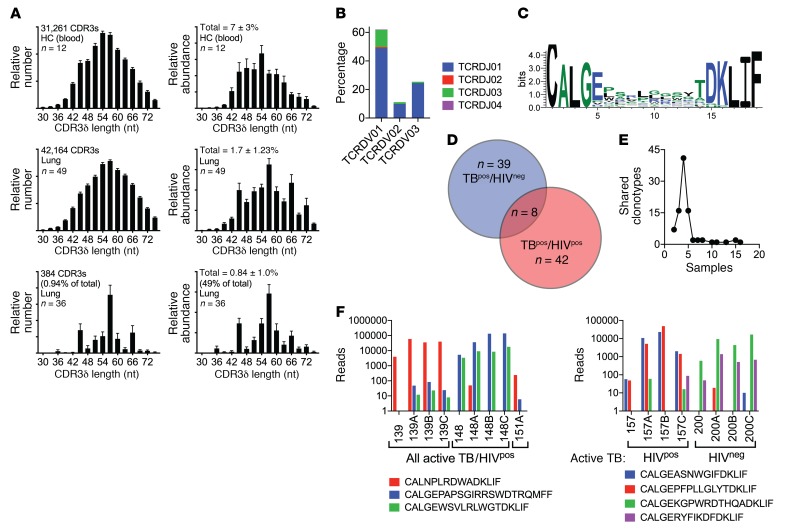
Enriched and abundant TCRδ clonotypes. (**A**) Distribution of CDR3δ lengths based on the relative frequency (left) or abundance (right) of all unique clonotypes in blood (top row), lung (middle row), or 92 enriched and abundant clonotypes in the lung (bottom row). Data represent the mean ± SEM. (**B**) Pairing of Vδ1, Vδ2, or Vδ3 with Jδ1, Jδ2, Jδ3, or Jδ4 gene segments for 92 lung-enriched and abundant TCRδ clonotypes. (**C**) CDR3δ motif for TCRδ clonotypes with a CDR3δ length of 19 aa. (**D**) Distribution of the 92 TCRδ clonotypes between HIV^neg^ or HIV^pos^ subjects with active TB. The numbers indicate unique clonotypes. (**E**) Sharing of unique CDR3δ DNA rearrangements among the 88 blood and lung samples that encoded each unique clonotype. (**F**) Shared clonotypes between different subjects and their relative abundance. Data represent the mean ± SEM.

To ascertain whether the expanded TCRδs were overrepresented because of a specific response or a stochastic process, we measured the distribution of CDR3δ lengths in blood and lung (Supplemental material). The blood and lung CDR3δ length distribution was similar, with a peak of 54–57 nucleotides (Figure 8A, left). In contrast, the 92 enriched and abundant clonotypes had skewed CDR3δ lengths with discrete peaks at 45, 57, and 66 nucleotides. Reanalysis of the distribution of CDR3δ lengths on the basis of abundance revealed a more pronounced skewing for the 92 enriched and abundant lung TCRδs (Figure 8A, right). Although these 92 clones only represent 1% of the unique lung TCRδ clonotypes, they account for half of all γδ T cells detected among the 49 lung samples. The Vδ and Jδ genes used by these 92 TCRs were biased and used Vδ1Jδ1 and Vδ1Jδ3 more frequently compared with blood (compare Figure 8B with Figure 6E). We determined whether a motif was present for CDR3δs with a length of 57 nucleotides. We found that leucines and prolines were modestly enriched, but no clear motif was evident (Figure 8C).

The 92 TCRδs were similarly distributed among HIV^neg^ or HIV^pos^ subjects with active TB (Figure 8D). We next determined whether there was sharing of unique TCRδs between subjects and found that there was less sharing than for the GEMs or MAITs (Figure 3, Figure 4, and Figure 5). Most TCRδ clonotypes were present in 1–4 samples, which corresponds to the number of samples per individual, although we detected 1 TCRδ clonotype in 16 samples (Figure 8E). Evaluation of the TCR DNA rearrangements revealed that a single recombination event encoded each TCRδ clonotype in most cases. When more than 1 unique DNA rearrangement produced the same aa sequence, a single rearrangement was dominant, and the others were detected at an extremely low frequency. Careful examination of these DNA sequences showed them to differ primarily in the 3′ region of the Vδ gene, consistent with PCR errors, but these sequences did not represent a distinct CDR3δ rearrangement (Supplemental Figure 5). However, we detected sharing of certain clonotypes between individuals (Figure 8F).

**Figure 9:**
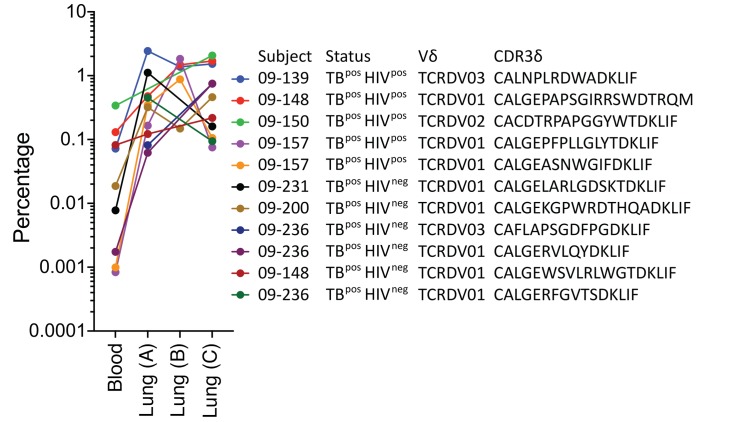
Enriched and abundant TCRδ clonotypes. The 11 TCRδ clonotypes with the highest frequencies in the lungs of subjects with active TB. The frequency of each clonotype in the blood and lungs is shown, along with the subject number, status, Vδ gene, and CDR3δ aa sequence.

Finally, although the subjects often had more than 1 expanded TCRδ clonotype in the lungs, generally 1 clonotype was dominant. Many of the 92 δTCR clonotypes had undergone major expansions in the lungs compared with the blood, and several were enriched by 10- to 2000-fold (Figure 9). Four of the clonotypes accounted for more than 1% of the total productive TCRs in the lung. Of the 11 most abundant clonotypes, only 1 used the Vδ2 gene, 2 used Vδ3, and the remaining 9 used Vδ1. Thus, certain γδ T cells, with bias for Vδ1, undergo dramatic clonal expansion in the lungs during active TB infection and bear a greater similarity to conventional T cell responses than to DURTs.

## Discussion

In this study, we took a novel approach to investigating human immunity to TB infection by analyzing the DURT and γδ T cell response via TCR repertoire sequencing. Importantly, we validated this approach by demonstrating a close correlation between the frequency of MAITs and γδ T cells identified by flow cytometry and the abundance of MAIT and γδ TCR clonotypes in the same PBMC samples. Using this technique, we were thus able to perform a comprehensive and unbiased characterization of the DURT and γδ T cell repertoire in blood and matched lung tissue from TB-infected individuals. Our ability to analyze TB-infected human lung tissue is important, as, to our knowledge, this is the first report to directly examine these DURT subsets at the site of disease in humans. A limited number of studies have examined specific DURT subsets within bronchoalveolar lavage (BAL) fluid, but the relationship between T cells within the bronchoalveolar space and the lung parenchyma is unclear (40). Moreover, the complexity of examining individual DURT subsets, using tetramers, for example, has meant that studies tend to focus on one specificity. In addition, T cell activation can lead to downregulation of surface markers that play a role in the identification of DURT subsets. MAITs, for example, are typically identified by dual surface expression of Vα7.2 TCR (TRAV1-02) and CD161, but this latter molecule is downregulated during TCR activation (41). Likewise, iNKTs are defined using tetramers or antibodies that bind the canonical iNKT Vα24^+^Vβ11^+^ TCR, which is downregulated when iNKTs encounter their cognate antigen. The presence of the canonical DURT TCR DNA rearrangement is not affected by the cell activation state and thus, we believe, presents an unbiased view of T cell frequency.

One possible example of TCR downregulation as a confounder of T cell enumeration is illustrated by examining iNKT cell frequency. Several studies relying on flow cytometry have reported a reduction of iNKTs in the blood of individuals with active Mtb or HIV infection compared with that of HCs or individuals with latent infection (42–45). Our sequence data, however, showed that the frequency of the iNKT TCR in blood was not significantly affected by TB or HIV-TB coinfection. It is possible, therefore, that the observed reduction of iNKTs during active TB and HIV could relate, at least in part, to TCR downregulation following antigen encounter. This is supported by evidence of recent TCR triggering on the remaining iNKTs detected by flow cytometry during active TB (43, 45). In addition, we found no evidence that iNKTs were relocating to the lungs, an idea that has been put forward to explain their loss from circulation during active disease (42).

In contrast, the reported loss of circulating MAITs in active TB (46–49) is supported by our TCR data, which showed a significant depletion in both TB- and HIV-TB–coinfected individuals. The depletion of circulating MAITs has been hypothesized to be a consequence of MAIT cell recruitment to the lungs (8), and T cells positive for the Vα7.2 TCR (TRAV1-02) were detected in histological sections of TB-infected human lung tissue (46). However, data on their frequency in TB-infected lungs are lacking, and the impact of HIV on these cells is unknown. HIV results in a profound loss of MAITs from both the circulation and the gut mucosa, a loss that is not restored by antiretroviral therapy (ART) (30, 50). Similarly, SIV infection of NHPs is associated with a reduction of MAITs in the blood and BAL fluid (39). Therefore, it is hypothesized that HIV also causes a loss of lung-associated MAITs that could contribute to increased TB susceptibility (31). As with iNKTs, we believe our data offer the first unbiased look at the impact of TB and HIV-TB infection on the frequency of MAITs within the human lung. On the basis of the experimentally defined MAIT sequences (MAIT_EXT_) and an extended definition of MAIT TCRs (MAIT-like), we found no evidence of accumulation of MAITs in lung tissue during active TB or of the depletion of these cells in HIV-TB–coinfected individuals. Indeed, the only group with significantly different MAIT frequencies was the group of HIV-infected individuals with prior TB infection, who possessed more MAITs than expected. Although we could not identify clinical features that might explain this MAIT enrichment, the observation strongly suggests that HIV infection, per se, does not deplete MAITs from the human lung.

The third DURT subset we examined, the group 1 CD1–restricted T cells, has not been extensively studied in TB-infected or HIV-TB–coinfected individuals, in part because of the low frequency of these cells and the lack of phenotypic markers for their detection. Enzyme-linked immunospot (ELISpot) assays revealed that CD1b-restricted T cells recognizing mycolic acid (MA) were enriched in the blood and BAL fluid of subjects with active TB compared with that of HCs (51). CD1b and GMM tetramer–staining T cells, which would include GEMs, were detected in the blood of subjects with active TB, but not in that of individuals with HIV-TB coinfection (33), although the frequency in TB-uninfected individuals was not reported. Here, we show that, like MAITs, GEMs were highly depleted from the blood of individuals with TB infection or HIV-TB coinfection but were not enriched in the lungs of these individuals. Indeed, GEMs were found to occur at a much lower frequency in the lung than were the other DURTs studied and were not detectable in some individuals. It is possible that GEMs, or indeed any of the DURTS investigated here, were elevated at certain stages of TB infection, either in the blood or the lung, but we found no evidence to support this in our chronically infected individuals. Of note, as both MAIT and GEM TCRs were depleted from the blood of all TB-infected subjects, our data suggest that these DURT subsets respond differently to TB and HIV-TB infection compared with iNKTs, which were not depleted.

These data also provide the opportunity to examine subtle differences in the DURT repertoire in a way that is not readily possible by conventional approaches. Unlike iNKTs, which are defined by an entirely invariant TCR, MAITs and GEMs express semi-invariant TCRs that display differences in antigen reactivity (16, 37, 52). This could lead to the preferential expansion of certain clonotypes in response to specific infections or tissue locations. We found no evidence of skewing of the MAIT or GEM TCR repertoire, either in the blood of diseased individuals versus HCs, or in the blood versus lungs of subjects with TB. However, the observation that several TCRs had GEM-like CDR3α sequences that were not derived from the canonical TRAV01-2 chain was unexpected. The selection of identical CDR3αs from different recombination events suggests that these TCRs are preferentially expanded in vivo, presumably because of antigen encounter. The GEM clone CAVLNTGGFKTIF, for example, is known to react with MA, an essential long-chain mycobacterial lipid, but not with the related Mtb antigen GMM (37). The TRAV01-2 recombination originally described for this CDR3α was detected in only 6 of 12 blood samples from HCs and in 6 of 49 lung samples from patients with TB, in whom it occurred at very low frequency. However, the CDR3 sequence itself was present at a higher frequency in the blood of all HCs and in most TB-infected blood (25 of 27) and lung (38 of 49) samples. This therefore confirms that MA-reactive GEMs are in fact present in TB-infected lung tissue, but they would not have been detected on the basis of their conventional definition or by flow cytometry using TRAV01-2 antibodies (37). Likewise, the GMM-reactive CDR3α CAVRNTGGFKTIF was present in the blood and lungs of most TB-infected subjects, but primarily through non–TRAV01-2 rearrangements. Importantly, overall we found no evidence of differences in DURT antigen specificity between blood and lung tissue, as previously described for conventional Trms (53, 54).

In addition to DURT T cell subsets, this study provided a unique opportunity to examine the γδ T cell repertoire in human lung tissue. There is growing evidence that γδ T cells play a protective role in Mtb infection, particularly those γδ T cells expressing the Vγ2Vδ2 (also called Vγ9Vδ2) TCR. BCG vaccination expands this subset in both adults and infants (55–57), and these T cells can directly kill Mtb-infected target cells and enhance conventional T cell immunity (21, 58). Moreover, Vγ2Vδ2 T cells are able to recognize immunogenic phosphoantigens through a novel “inside-out” mechanism involving butyrophilin 3A1, which can facilitate the sensitive detection of intracellular pathogens including Mtb (59). Importantly, the Vγ2Vδ2 T cell subset exists only in humans and NHPs, but not in mice, which has limited their study (5). The peripheral blood TCRδ repertoire in humans is dominated by δ2-expressing T cells, which constitute 60%–90% of γδ T cells in circulation, and the majority of which are from the Vγ2Vδ2 T cell subset (60, 61). Our TCR sequence data agree, as the Vδ2 chain comprised approximately 60% of the TCRδ repertoire in the blood of HCs. Using an anti–δ chain antibody, we detected an increase in γδ T cells in the blood of HIV^neg^ subjects with microbiologically confirmed pulmonary TB compared with HCs and subjects with bacterial pneumonia or other lower respiratory tract infections (62). Similar observations were made using Vδ1 and Vδ2 TCR–specific antibodies (60); and, more recently, an expansion of γδ T cells detected by flow cytometry was reported in household contacts of individuals with TB (63), supporting a potential role for these cells in response to primary infection. However, active TB has also been linked to a loss of circulating effector Vγ2Vδ2 T cells (64), and a progressive loss of these cells correlates with pulmonary disease severity (65). The reason for these disparate results is not clear, but the data presented here clearly support a specific loss of Vδ2 TCRs from the blood of subjects with severe TB, and no overall increase in TCRδ frequency. This is also consistent with previous work showing depletion of Vγ2Vδ2 T cells from the blood in chronic HIV infection, with limited restoration by ART (66, 67).

As with DURTs, one possible explanation for the loss of Vδ2 TCRs from the blood could be recruitment to the lungs. Experimentally, a large expansion of γδ T cells expressing Vδ2 was observed in both the blood and BAL fluid of NHPs 5 weeks after intravenous injection of BCG (23, 58). Aerosol infection of NHPs with H37Rv, however, only induced expansion of Vγ2Vδ2 T cells in the lungs (up to 23% of all CD3^+^ T cells) and not in the blood, suggesting that the route of infection is key (62, 68). The involvement of γδ T cells, and Vγ2Vδ2 T cells specifically, at the site of disease in humans has not, however, been well studied. TB-reactive γδ T cells were found to be severely depleted in BAL fluid of subjects with active TB compared with that of HCs or of subjects with non-TB lung granulomatous disease (69); and Vδ2 T cells were significantly less frequent in the BAL fluid of subjects with active TB compared with matched blood (70). Taken together, these limited studies broadly support our own findings, in that the overall frequency of TCRδs was not different between the blood and the lungs, but the frequency of Vδ2 TCRs in general was much lower in the lung than in the blood. Moreover, the loss of Vδ2 T cells in the blood was not associated with a reciprocal expansion in the lung in subjects with progressive TB.

It is interesting that Vδ1 dominates the lung γδ repertoire, as this chain is found at a relatively low frequency in the blood (71). An enrichment of Vδ1 in the mucosal epithelium of the intestines, lungs, and genital tract has been described previously (71, 72), suggesting a role for these cells at barrier sites. In mice, specialized intraepithelial lymphocytes (IELs) exclusively express the Vδ1 TCR and populate epithelial barriers early during development (73). Vδ1^+^ T cells are activated by various ligands, including stress-induced self-antigens and glycolipids presented by CD1c or CD1d and others that remain unidentified (74). Functionally, Vδ1^+^ T cells play different roles, depending on the disease context. They are the dominant subset in breast tumors and can actively suppress effector T cell functions and block DC maturation (75, 76). Given that γδ T cell infiltration is directly correlated with tumor progression and poor patient outcome, this infiltration could be important (77). Vδ1^+^ T cells also accumulate in the synovial fluid of subjects with Lyme arthritis, whereby these cells limit CD4^+^ T cell responses against the causative agent *Borrelia* *burgdorferi* (78). Finally, Vδ1^+^ T cells are an important source of IL-17 during the response to lung bacterial infection in murine models (79). Given the abundance of Vδ1 T cells, understanding their function in the TB-infected human lung will be important.

Perhaps the most surprising observation revealed by this study was the impressive lack of overlap between γδ T cells in the blood and lungs. The shift in dominance from Vδ2 in the blood to Vδ1 (and Vδ3) in the lungs was a broad difference that could be observed through flow cytometry. However, the finding that most of the TCRδs were unique to the blood or lungs only became apparent through sequencing. Moreover, within the same lung, the separation of the TCRδ repertoires between different tissue sections was remarkable. The apparent propensity of γδ T cells to establish Trm populations in nonlymphoid organs and mucosal barriers (6) may explain the lack of overlap between blood and lung. However, the heterogeneity across lung tissue was surprising and suggests that γδ T cells undergo highly localized expansions as they encounter antigen. There are several important possible implications of this observation. First, and unlike αβ DURTs, there is no reason to expect that the antigen specificity of γδ T cells detected in the blood will match that of γδ T cells in lung tissue. This may be of relevance for studies seeking γδ T cells correlates of protection in the blood, or when assessing potential future vaccine candidates (11). Second, direct evidence from NHPs and indirect evidence from histological examination of human TB-infected lung tissue suggests that TB immune control in the lungs is highly heterogeneous, as both controlling and progressing TB lesions can be simultaneously observed within the same lung (18, 80). Since we now know that the γδ T cell repertoire is also extremely heterogeneous with the lung, it is plausible that TB granuloma progression could be influenced by localized expansions of γδ T cells. Our identification of highly expanded and enriched or unique lung TCRδ clonotypes supports this hypothesis. Single-cell sequencing of γδ T cells isolated from individual TB granulomas could further test this idea. Locally expanded γδ T cells could then be cloned and screened for Mtb reactivity and ultimately be used to identify potential γδ T cell vaccine targets (11).

In summary, these data represent a first attempt to describe the unconventional T cell landscape of human TB–infected lung tissue using high-throughput TCR sequencing. This allowed us to address several questions about the recruitment of Mtb-reactive DURTs into the human lung during TB and to assess the impact of HIV coinfection on those cells. In one sense, the relative preservation and even distribution of DURTs within the lung, despite HIV coinfection, validate the efforts to elicit these cells with novel vaccine strategies. In contrast to DURTs, the extreme discrepancy between the TCRδ repertoire in blood versus lung and the heterogeneity of this repertoire at the site of TB disease are striking. Whether these highly localized differences contribute to TB control at the granuloma level remains to be determined, but these data suggest that repertoire sequencing is a powerful starting point to address these important questions. The highly tissue-resident nature of these γδ T cells, as suggested by the clear compartmentalization between blood and lung, may represent a unique opportunity to elicit long-lived and protective Trms at the site of disease.

## Methods

### Participants.

TB-infected lung tissue and blood were obtained from participants undergoing medically indicated lung resections to treat active TB or TB sequelae, including hemoptysis, bronchiectasis, shrunken or collapsed lung, or nonresponsive infection at the King Dinuzulu Hospital and Inkosi Albert Luthuli Central Hospitals in Durban, KwaZulu-Natal. TB-negative control samples were obtained from healthy tissue margins from lung cancer resections. Blood from non–TB-infected controls was obtained from a healthy volunteer cohort at the King Edward Hospital in Durban.

### PBMC isolation.

Blood was collected in BD Vacutainers (sodium heparin, BD), PBMCs were isolated using Ficoll-Histopaque (MilliporeSigma) density gradient centrifugation and cryopreserved in freezing media (10% DMSO; 90% FCS) until needed. All PBMC samples were frozen before use.

### Flow cytometry.

PBMCs from were thawed in R10 supplemented with DNaseI, washed twice by centrifugation (500 *g*) and rested for 2 hours at 37°C, 5% CO_2_, followed by centrifugation and resuspension of the pellet in 50 μL antibody mixture for 20 minutes at room temperature. Cells were washed twice (PBS) and fixed with 2% PFA. Cells then acquired using an Aria Fusion Cytometer (BD) and analyzed with FlowJo Software, version 9.9.6 (Tree Star). The antibody cocktail consisted of LIVE/DEAD Fixable Near-IR Dead Cell Marker (Invitrogen, Thermo Fisher Scientific); anti–CD3 Brilliant Violet 785, clone OKT3; anti–γδ TCR PE, clone B1; anti–Vδ2 TCR PerCpCy5.5, clone B6; anti–Vγ9 TCR APC, clone B3; anti–Vα7.2 BV711, clone 3C10; anti–CD26 PE-Cy5 clone BA5b; anti–CD161 Brilliant Violet 605, clone HP3G10 (all from BioLegend); anti–CD45 V500, clone HI30 (BD); and anti–Vδ1 TCR FITC clone TS8.2 (Thermo Fisher Scientific).

### Lung processing.

For each lung, different areas were removed by the operating surgeon and corresponded to the surgeon’s assessment of the most diseased (A), intermediate (B), and healthiest tissue (C) tissue on the basis of experience and the preoperative radiological data.

### DNA extraction.

For lung, tissue was added to a 2-mL tube containing 100 μL Zarconia beads (BioSpec) and bead-beaten 5 times (7000 rpm for 60 seconds), with 30 seconds on ice between intervals using MagNA Lyser (Roche Diagnostics). DNA was extracted from snap-frozen lung tissue and PBMCs using a DNeasy Blood and Tissue Kit (QIAGEN) according to the manufacturer’s instructions.

### Next-generation sequencing and analysis.

For TCRα sequencing, purified genomic DNA was sequenced by Adaptive Biotechnologies using the ImmunoSEQ assay (http://www.immunoseq.com) as previously described (25). PBMCs were subjected to deep-resolution sequencing (identified TCRs with a frequency of 1 in 2 × 10^5^ to 1 × 10^6^), and lung samples were subjected to survey resolution (1 in 60,000), the rationale being that survey-level sequencing was sufficient for identification of expanded TCRs in the lung, but a greater sequencing depth might be required to identify these TCRs if they were less expanded in blood. Data were analyzed using the ImmunoSEQ analyser tools. TCR clonality is a metric related to Shannon’s Diversity Index, which measures the diversity and abundance of a cell population (81, 82). Productive rearrangements (in-frame without stop codons) and TCR gene segment assignment were done as part of the ImmunoSEQ assay. Throughout our analysis, we applied the following filters: frame = in and reads >1. In most of our analyses, we stated TCR frequencies as a percentage of total productive rearrangements. In the violin plots, the metric of productive TCR frequency, as a fraction of 1, was used. MAIT Match Server (http://www.cbs.dtu.dk/services/MAIT_Match/) was used to identify additional TCRs with MAIT-like features (16). BioVenn (http://www.biovenn.nl) was used to compare overlapping sets of TCRs (83), and CDR3 motifs were generated using WebLogo 3 (http://weblogo.threeplusone.com) (84).

### Statistics.

Data are presented as the mean ± SEM. A 2-tailed Student’s *t* test was used for normally distributed data for comparisons of 2 groups. A 1-way or 2-way ANOVA was used for comparison of more than 2 groups, followed by Bonferroni’s or Sidak’s post test. A *P* value of less than 0.05 was considered statistically significant. Statistical and graphical analyses were performed using GraphPad Prism, version 8 (GraphPad Software).

### Study approval.

All participants provided informed consent, and the study was approved by the Biomedical Research Ethics Committee (BREC) of the University of KwaZulu-Natal (BE019/13 for the lung cohort and BE 037/13 for the healthy volunteers).

## Author contributions

PO conducted experiments and assisted with data analysis and writing of the manuscript. AJCS established the lung cohort. FK, KJD, IA, and RM obtained samples and analyzed clinical information. SB and AL are co–senior authors who designed and implemented this study, analyzed the data, and cowrote the manuscript with PO.

## Supplementary Material

Supplemental data

## Figures and Tables

**Table 1:**
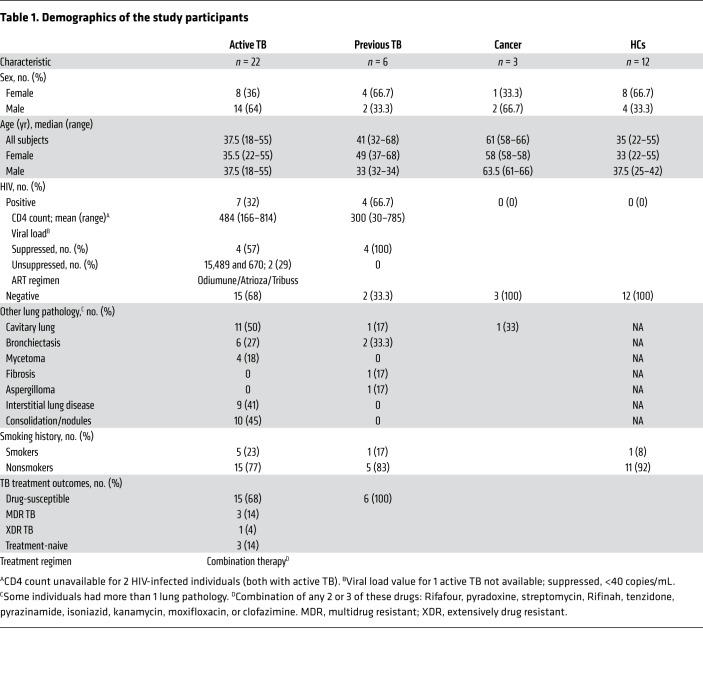
Demographics of the study participants

**Table 2:**
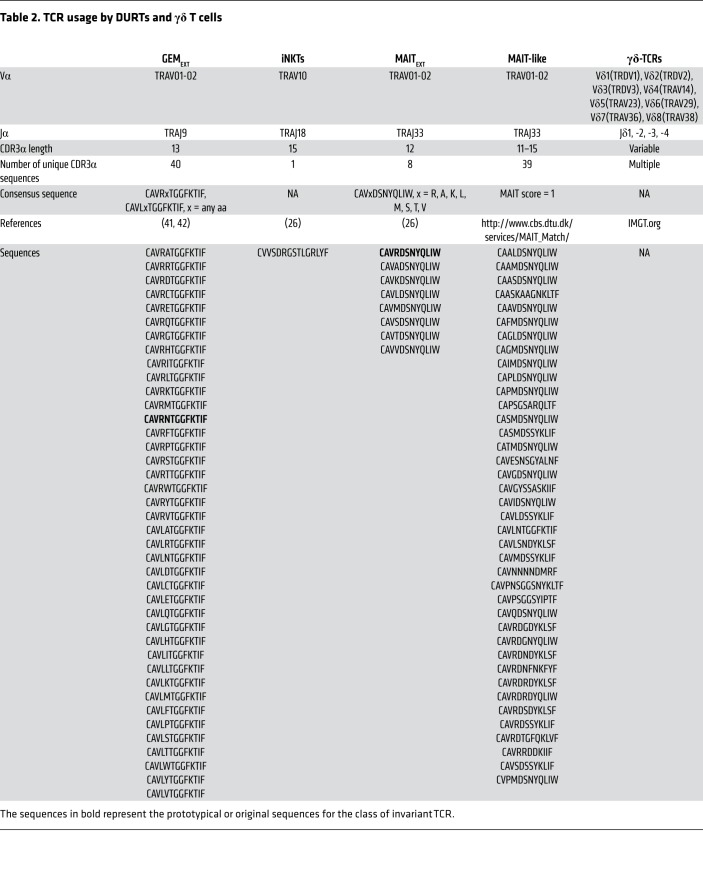
TCR usage by DURTs and γδ T cells

## Version 1 11/25/2019

Electronic publication

## Version 2 01/02/2020

Print issue publication
