# Supplementary material for: Differential skewing of donor-unrestricted and γδ T cell repertoires in tuberculosis-infected human lungs
Source: J Clin Invest. 2019 Nov 25;130(1):214–30. doi: 10.1172/JCI130711 (PMC6934215; doi:10.1172/JCI130711)
Supplement: Supplemental data [file jci-130-130711-s104.pdf]

Supplemental information

Nomenclature

The nomenclature used in this paper adheres to the rules established by The International Immunogenetics Information System (IMGT, http://www.imgt.org), and adopted by the HUGO Gene Nomenclature Committee (ref: https://doi.org/10.3389/fimmu.2014.00022). As we deal exclusively with the TCR loci alpha and delta, these rules can be summarized as follows:

1. The first three letters (i.e., "TRA" or "TRD") specify the T cell receptor alpha or delta locus
2. The fourth letter (i.e., "V" or "J") specify whether it is a V-gene or a J-gene
3. The numbers unambiguously identify the particular gene

Clonality

Clonality is a calculated number that measures the diversity of the T cell receptor repertoire. It considers both the number of unique TCRs as well as their relative abundance. Thus, if every TCR is unique (i.e., a maximally diverse repertoire), the clonality score would be "0". On the other hand, if the TCR repertoire were dominated by a single TCR rearrangement (i.e., a monoclonal repertoire), the clonality score would be "1" bias. In general, the clonality scores of the TCR repertoire of peripheral blood from healthy controls is very low (see Figure 1d). Clonality is calculated as 1-(entropy/log2[# unique TCRs]), with entropy representing a measure of diversity within a complex data set, which is also known as the Shannon-Wiener index, Shannon's diversity index or Shannon's entropy [PMID: 9519765, PMID: 23771160].

CDR3

The CDR3 region is defined as the amino acids between the highly conserved amino acids 104 (CYS in V-region) and amino acid 118 (PHE or TRP in J-region). In this manuscript, we use CDR3 interchangeably with "junction," which is inclusive of the two conserved amino acids, and is technically two amino acids longer than the CDR3. The CDR3 regions of the different TCR chains have their own characteristic length distributions. Skewing of the typical distribution (i.e., as seen in healthy controls) can indicate a bias in the expansion of certain T cell clonotypes. Most understandably, this can occur in malignancies involving the lymphoid system, where there is a monoclonal expansion of a malignant T or B cell. However, it can also occur during specific adaptive immune responses, in which case the expansions tend to be polyclonal. In contrast, a process that involves polyclonal activation, or a stochastic process, would not be predicted to alter the CDR3 distribution.

Supplemental Figures 1-5 (below)

Supplemental Figure 1. CD1-restricted clonotypes

A. TCRs identified

**T cell line**	CD8-1	CD8-2	LDN5
**restriction**	CD1c	CD1a	CD1b
**specificity**	phospholipid	lipid	glucose monomycolate
**CDR3α length**	13 aa	12 aa	12 aa
**TRAJ**	TRAJ17	TRAJ34	TRAJ09
**CDR3α aa**	**RRAAGNKLTF**	**RHNTDKLIF**	**MYTGGFKTIF**

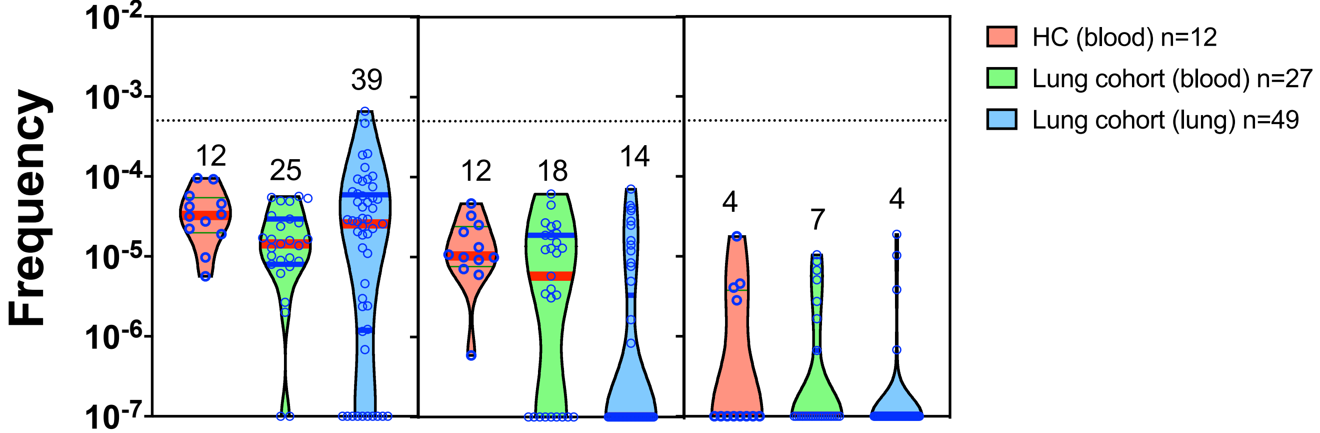

B. TCRs not found

**T cell line**	DN1	DN.POTT
**restriction**	CD1b	CD1b
**specificity**	mycolic acid	mycolic acid
**CDR3α length**	16 aa	13 aa
**TRAJ**	TRAJ57	TRAJ31
**CDR3α aa**	PLSLPGGSEKLVF	RDEGWARLMF

Five well-characterized group 1 CD1-restricted T-cells have had their antigens defined, their CD1 restriction determined, and have been sequenced (38,39).

A. We detect three of their CDR3α sequences among the TCRs expressed in our lung cohort. The name of the T-cell line, the antigen presenting molecule, the antigen specificity, their CDR3α length, TRAJ segment, and CDR3α sequences are shown. Violin plots show that frequency and the number of individuals in which the three CDR3α sequences were detected. Lines: red bar, median; blue bars; quartiles. The dotted line represents a frequency of 0.0005, or 0.05% of all productive TCRα rearrangements.

B. We searched for but did not find CDR3α sequences used by two additional CD1-restricted T-cell lines.

Supplemental Figure 2. GEMs clonotype frequencies

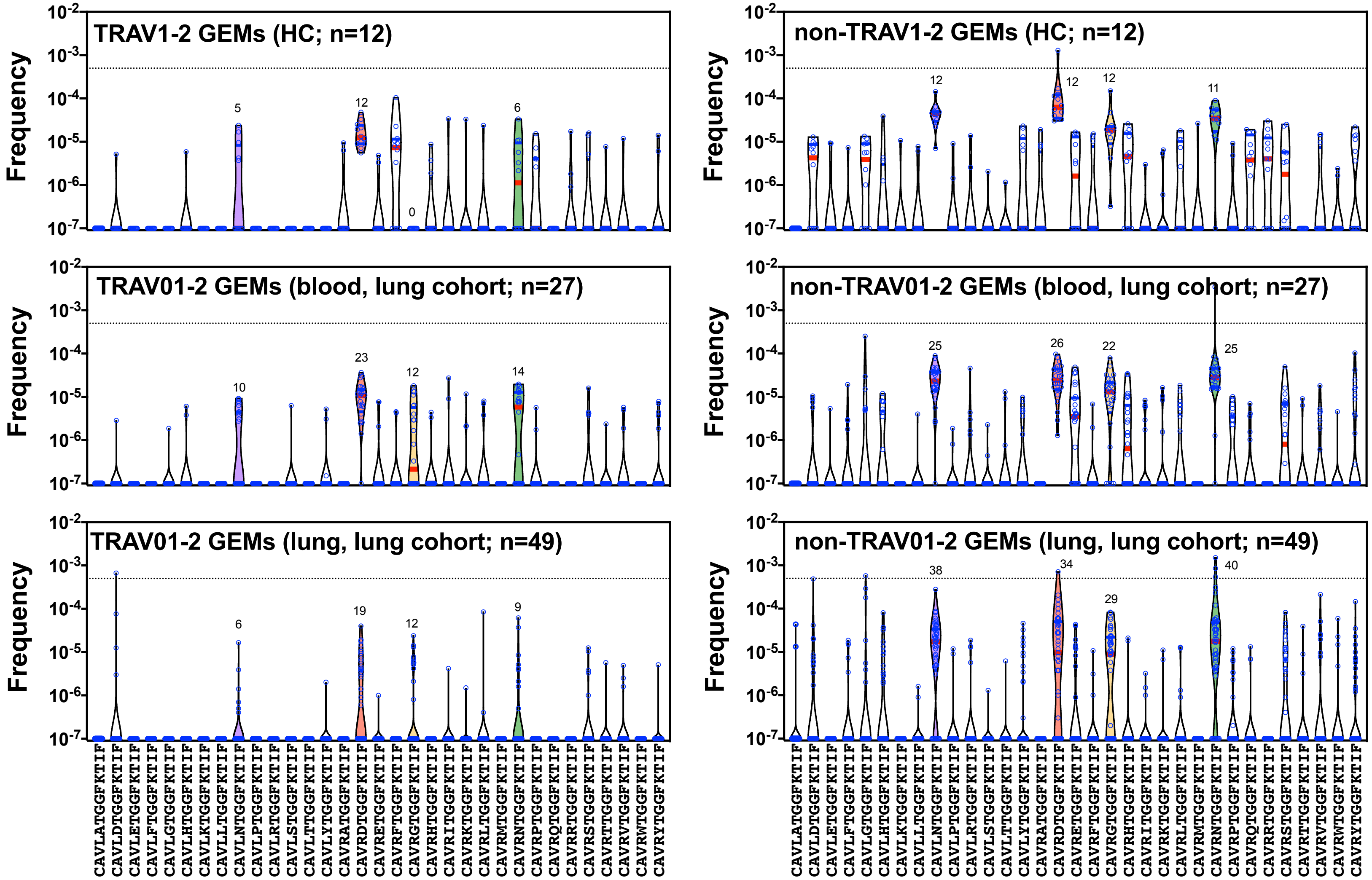

Clonotypes that had CDR3α with the aa sequence CAV(R/L)xTGGFKTIF were identified in healthy controls (blood, 1st row, n=10), and the lung cohort (blood, 2nd row; n=27) or lung (3rd row, n=49). The frequencies of clonotypes that used TRAV01-2 are shown in the first column (left), while the frequencies of clonotypes that use Vα genes other than TRAV01-2 (i.e., non- TRAV01-2) are shown the second column (right). The numbers on top of some columns indicates in how many subjects was the CDR3α sequence detected. For samples for which the indicated clonotype was not detected, the frequency was assigned 0.0000001. The dotted line corresponds to a productive frequency of 0.05%. The colored violins indicate the clonotypes that were most frequently detected in lung. Lines: red bar, median; blue bars; quartiles.

Supplemental Figure 3. MAIT-like clonotypes

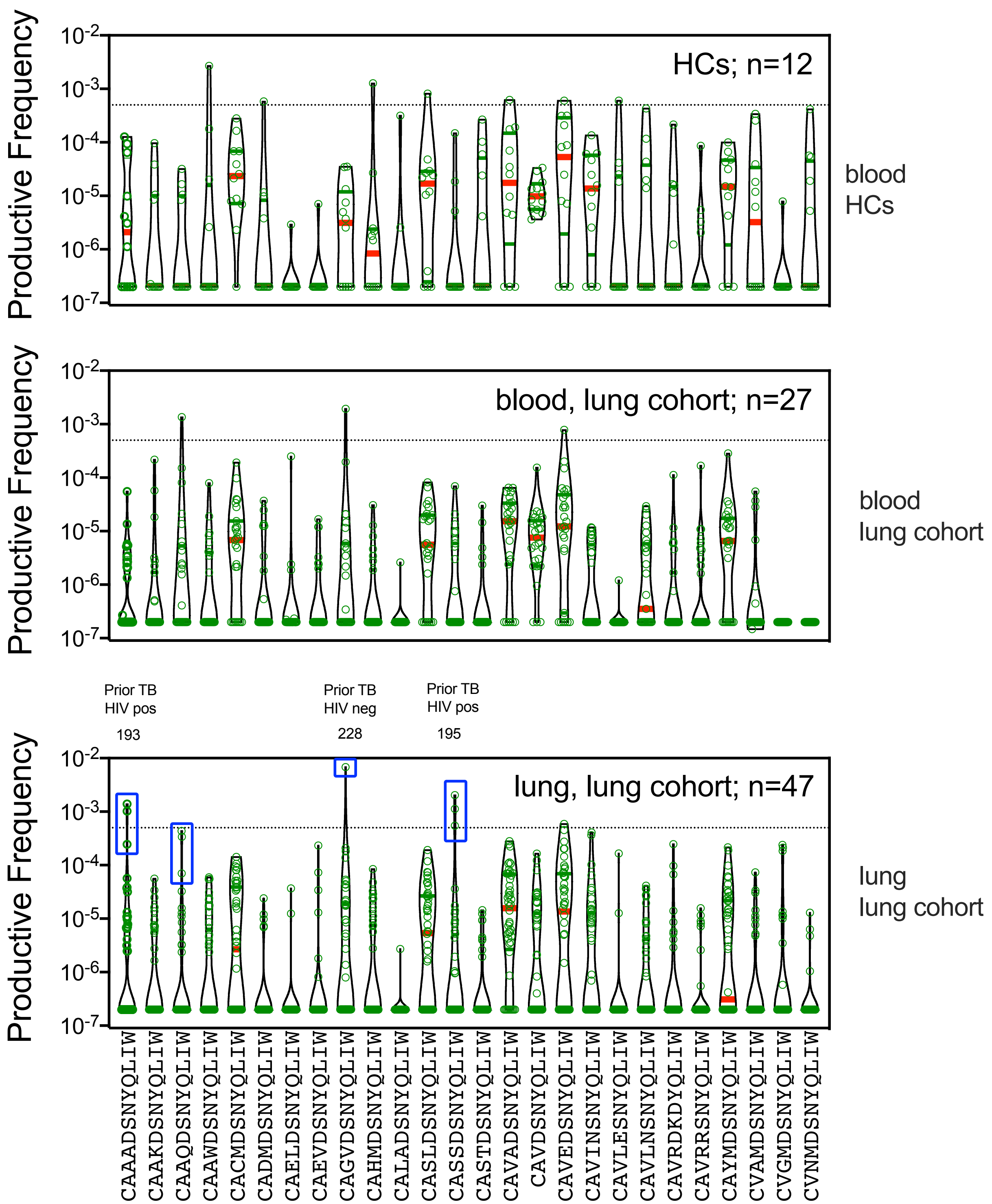

MAIT-like TCRs with a MAIT match score between 0.950 – 0.999 were identified among the TCRs from all of our samples. We identified 600 additional MAIT-like TCRs, which used Va1-02 and Ja33. From these, only 43 had a sum total frequency (the sum of their frequencies from all the samples) greater than 0.02% (or 1 in 5000 T cells). We calculated the individual frequencies for each of these 43 TCRs among all of the samples. We identified 26 TCRs that had a frequency of at least 0.02% in at least one individual sample. These data are plotted above. Interestingly, the pattern is similar to what we identified for the other MAIT TCRs – namely that the largest expansions occurred in HIVpos individuals with a history of prior TB.

Supplemental Figure 4. Correlation between TRDV and TRDJ use

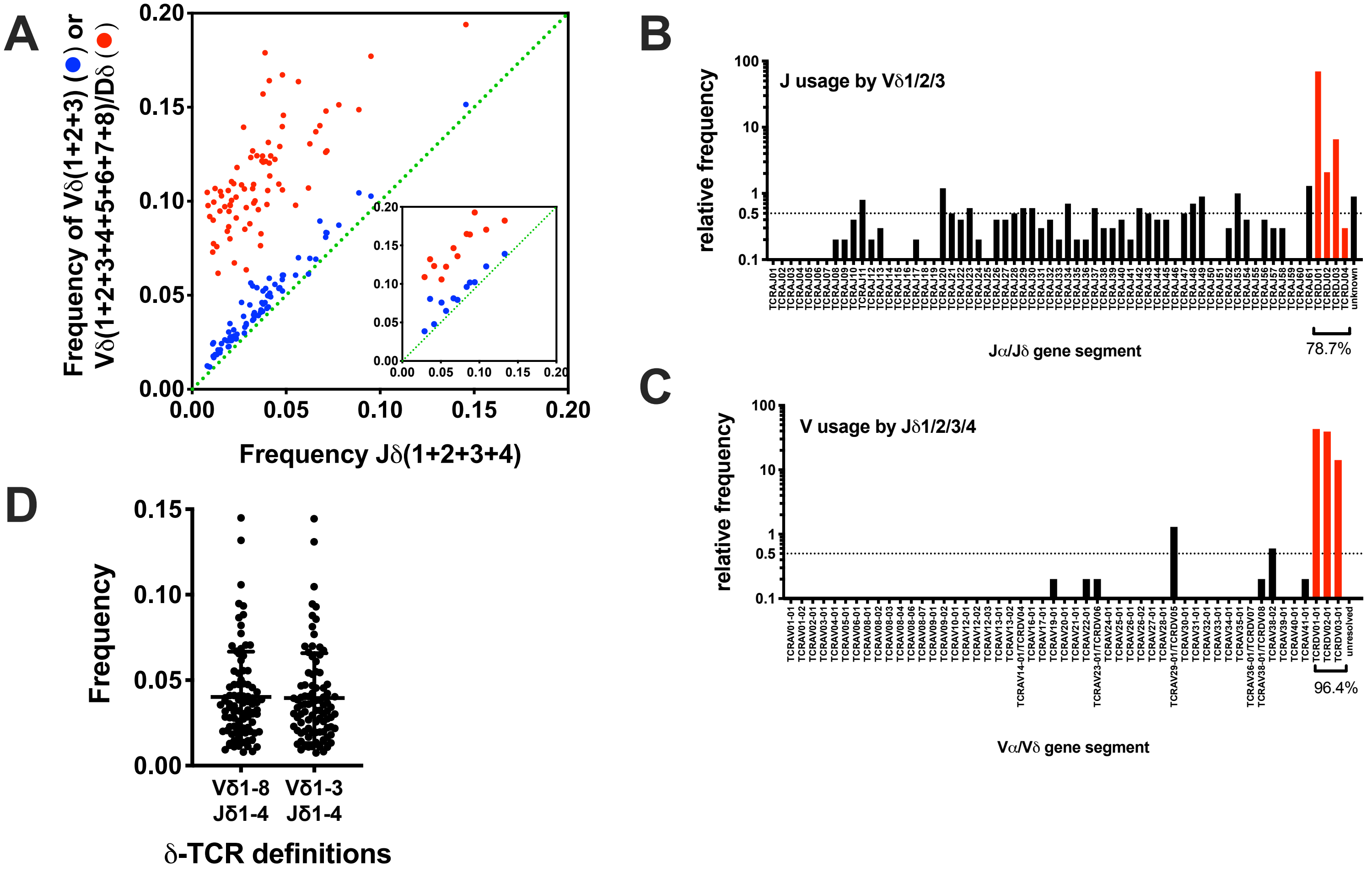

A. The correlation between the combined frequency of TRDJ1, TRDJ2, TRDJ3, or TRDJ4 gene segments per sample, versus the combined frequency of TRDV1, TRDV2, or TRDV3 gene segment (blue; R^2 =0.956) or the combined frequency of TRDV1, TRDV2, TRDV3, TRDV4/TRAV14, TRDV5/TRAV29, TRDV6/TRAV23, TRDV7/TRAV36, or TRDV8/TRAV38-2, which also use a TRDD gene segment (red; R^2=0.411). Inset, correlation of TRDV and TRDJ gene usage for the healthy control cohort (blood, n=10) R^2 =0.88 (blue) vs 0.76 (red). The dotted lines have a slope=1, for reference. Each dot represents an individual. The combined frequency of TRDV1, TRDV2, and TRDV3 V gene segment correlates best with the combined frequency of TRDJ1, TRDJ2, TRDJ3, or TRDJ4, because TCRs using TRDV4/TRAV14, TRDV5/TRAV29, TRDV6/TRAV23, and TRDV7/TRAV36 also are used as TRAV genes and recombine with TRAJ gene segments.

B. TRDJ usage by TCRs that used the TRDV1, TRDV2, or TRDV3 gene segments. 78.7% of TCRs using a TRDV1, TRDV2, or TRDV3 V gene segment, used the TRDJ1, TRDJ2, TRDJ3, or TRDJ4 J genes (in red).

C. TRDV use by TCRs that included the TRDJ1, TRDJ2, TRDJ3, or TRDJ4 genes. 96.4% of TCRs using a TRDJ1, TRDJ2, TRDJ3, or TRDJ4 J gene segment, also used TRDV1, TRDV2, or TRDV3 V-regions (red).

D. Two methods of determining the frequency of γδ T-cells within each tissue sample is compared. In the first column, the total frequency of γδ T-cells is calculated by including any TCR that uses TRDV1, TRDV2, TRDV3, TRDV4/TRAV14, TRDV5/TRAV29, TRDV6/TRAV23, TRDV7/TRAV36, or TRDV8/TRAV38-2, paired with TRDJ1, TRDJ2, TRDJ3, or TRDJ4. The second column defines a γδ T-cell as one that uses TRDV1, TRDV2, or TRDV3, paired with TRDJ1, TRDJ2, TRDJ3, or TRDJ4. After applying these two approaches to all of the samples we have analyzed (i.e., both the lung cohort and healthy controls), we find no difference between the two methods. These data led us to define a δ-TCR chain as one that uses TRDV1, TRDV2, TRDV3, TRDV4/TRAV14, TRDV5/TRAV29, TRDV6/TRAV23, TRDV7/TRAV36, or TRDV8/TRAV38-2, paired with TRDJ1, TRDJ2, TRDJ3, or TRDJ4, even though the contribution of TRDV4/TRAV14, TRDV5/TRAV29, TRDV6/TRAV23, TRDV7/TRAV36, and TRDV8/TRAV38-2, is negligible.

Supplemental Figure 5. Analysis of TCR DNA rearrangements

Rank (reads)	sample name	productive frequency	templates	reads	amino_acid	NA sequence
7	09-0148A	1.9889E-06	2	15	CALGEPAPSGIRRSWDTRQMFF	CAAAGTAC TTTGTGCTCTTGGGGAACCGGCCCCTTCGGGGATACGCCGCTCCTGGGACAC
12	09-0148B	1.66924E-06	2	15	CALGEPAPSGIRRSWDTRQMFF	CAAAGTAC TTTGTGCTCTTGGGGAACCGGCCCCTTCGGGGATACGCCGCTCCTGGGACAC
15	09-0148C	2.57627E-06	2	21	CALGEPAPSGIRRSWDTRQMFF	CAAAGTAC TTTGTGCTCTTGGGGAACCGGCCCCTTCGGGGATACGCCGCTCCTGGGACAC
14	09-0148C	7.36076E-07	2	6	CALGEPAPSGIRRSWDTRQMFF	ACAGTACTTTTGTGCGCTTGGGGAACCGGCCCCTTCGGGGATACGCCGCTCGTGGGACAC
11	09-0148B	6.67697E-07	1	6	CALGEPAPSGIRRSWDTRQMFF	AACGTACTTTTGTGCGCTTGGGGAACCGGCCCCGTCGGGGATACGCCGCTCCTGGGACAC
10	09-0148B	6.67697E-07	1	6	CALGEPAPSGIRRSWDTRQMFF	AAAGTCCTTTTGTGCGCTGGGGGAACCGGCCCCTTCGGGGATACGCCGCTCCTGGGACAC
1	09-0139A	1.95973E-05	2	48	CALGEPAPSGIRRSWDTRQMFF	AAAGTACTTTTGTGCTCTTGGGGAACCGGCCCCTTCGGGGATACGCCGCTCCTGGGACAC
2	09-0139B	3.28955E-05	3	84	CALGEPAPSGIRRSWDTRQMFF	AAAGTACTTTTGTGCTCTTGGGGAACCGGCCCCTTCGGGGATACGCCGCTCCTGGGACAC
3	09-0139C	9.27494E-06	1	24	CALGEPAPSGIRRSWDTRQMFF	AAAGTACTTTTGTGCTCTTGGGGAACCGGCCCCTTCGGGGATACGCCGCTCCTGGGACAC
4	09-0148	0.001303691	317	5357	CALGEPAPSGIRRSWDTRQMFF	AAAGTACTTTTGTGCTCTTGGGGAACCGGCCCCTTCGGGGATACGCCGCTCCTGGGACAC
5	09-0148A	0.004763947	1592	35929	CALGEPAPSGIRRSWDTRQMFF	AAAGTACTTTTGTGCTCTTGGGGAACCGGCCCCTTCGGGGATACGCCGCTCCTGGGACAC
9	09-0148B	0.014812187	5191	133104	CALGEPAPSGIRRSWDTRQMFF	AAAGTACTTTTGTGCTCTTGGGGAACCGGCCCCTTCGGGGATACGCCGCTCCTGGGACAC
13	09-0148C	0.016987772	5502	138473	CALGEPAPSGIRRSWDTRQMFF	AAAGTACTTTTGTGCTCTTGGGGAACCGGCCCCTTCGGGGATACGCCGCTCCTGGGACAC
16	09-0151A	2.37146E-06	1	6	CALGEPAPSGIRRSWDTRQMFF	AAAGTACTTTTGTGCTCTTGGGGAACCGGCCCCTTCGGGGATACGCCGCTCCTGGGACAC
6	09-0148A	2.38668E-06	2	18	CALGEPAPSGIRRSWDTRQMFF	AACGGACTTTTGTGCTCTTGGGGAACCGGCCCCTTCGGGGATACGCCGCTCCTGGGACAC
8	09-0148B	6.67697E-07	2	6	CALGEPAPSGIRRSWDTRQMFF	AAAGGACGTTTGTGCGCTTGGGGAACCGGCCCCTTCGGGGATACGCCGCTCCTGGGACAC
						******* ** ************** ***************** ********

TCRDV01	CAAAGTACTTTTGTGCTCTTGGGGAAC
TCRDJ03	CTCCTGGGACAC

3′ TCRDV01 | N, D, P sequences | 5′ TCRDJ03

A. Analysis of the CALGEPAPSGIRRSWDTRQMFF, which is the second most abundant CDR3δ based on its total abundance when the lung cohort was considered as a whole. 16 rearrangements were detected among four unique subjects (color coded), and in multiple samples per subject. Each sequence was rank based on abundance and clustered (which is the order shown). The productive frequency, template numbers, and reads is shown. The CDR3δ amino acid sequence is identical all (by definition). The DNA rearrangements varies. The sequences highlighted in yellow are all identical and are the dominant sequence in subject 09-148, but is also detected in subjects 09-139 and 09-151. The three sequences highlighted in orange are all identical, but appear to have an PCR error or sequence call error as there is a one nucleotide gap in the 3′ region of TRDV03. The remaining sequences (not highlighted), are all unique and the differences from the consensus sequence are indicated by the red font. Note, that except for a single sequence, all of the differences are in regions of the 3′ V or 5′ J, and hence are most likely to be PCR errors since these are regions of the TCR that are germline encoded. This leaves a single sequence that has a sequence difference in the junctional sequence, and possibly represents an independent rearrangement but one cannot rule out that this is also a PCR error. The germline the 3′ TRDV01 and 5′ TRDJ03 is indicated for references.

Supplemental Figure 5. Analysis of TCR DNA rearrangements (continued)

Rank (reads)	sample name	productive frequency	templates	reads	amino_acid	NA sequence
7	09-0139B	1.56645E-06	2	4	CALNPLRDWADKLIF	CAGTAAG ACTGAAGACAGTGCCACTTACTACTGTGCCTTGAACCCCCTCCGCGACTGGGC
3	09-0139A	4.08278E-06	2	10	CALNPLRDWADKLIF	CAG AAGGACTGAAGACAGTGCCACTTACTACTGTGCCTTGAACCCCCTCCGCGACTGGGC
4	09-0139A	3.6745E-06	2	9	CALNPLRDWADKLIF	CAGTA GGACTGAAGACAGTGCCACTTACTACTGTGCCTTGAACCCCCTCCGCGACTGGGC
12	09-0139C	1.54582E-06	2	4	CALNPLRDWADKLIF	AGTAAGGACTGAAGACAGTGCCACTTACTTCTGTGCCTTGAACCCCCTGCGCGACTGGGC
11	09-0139C	1.54582E-06	2	4	CALNPLRDWADKLIF	AGTAATGTCTGAAGACAGTGCCACTTACTACTGTGCCTTGAACCCCCTCCGCGACTGGGC
8	09-0139B	1.56645E-06	2	4	CALNPLRDWADKLIF	AGTAAGGACTGAAGACAGGTCCACTTACTACTGTGCCTTGAACCCCCTCCGCGACTGGGC
5	09-0139A	1.63311E-06	2	4	CALNPLRDWADKLIF	AGTAAGGTCTGAAGACAGTGCCACTTACTAGTGTGCCTTGAACCCCCTCCGCGACTGGGC
1	09-0139	0.000720941	163	3934	CALNPLRDWADKLIF	AGTAAGGACTGAAGACAGTGCCACTTACTACTGTGCCTTGAACCCCCTCCGCGACTGGGC
2	09-0139A	0.024308469	3045	59539	CALNPLRDWADKLIF	AGTAAGGACTGAAGACAGTGCCACTTACTACTGTGCCTTGAACCCCCTCCGCGACTGGGC
6	09-0139B	0.013740908	1188	35088	CALNPLRDWADKLIF	AGTAAGGACTGAAGACAGTGCCACTTACTACTGTGCCTTGAACCCCCTCCGCGACTGGGC
9	09-0139C	0.015263459	1179	39496	CALNPLRDWADKLIF	AGTAAGGACTGAAGACAGTGCCACTTACTACTGTGCCTTGAACCCCCTCCGCGACTGGGC
13	09-0148A	6.62967E-06	1	50	CALNPLRDWADKLIF	AGTAAGGACTGAAGACAGTGCCACTTACTACTGTGCCTTGAACCCCCTCCGCGACTGGGC
15	09-0151A	9.56487E-05	10	242	CALNPLRDWADKLIF	AGTAAGGACTGAAGACAGTGCCACTTACTACTGTGCCTTGAACCCCCTCCGCGACTGGGC
14	09-0148A	6.62967E-07	1	5	CALNPLRDWADKLIF	AGTAAGGACTGAAGACCGTGCCACTTACTACTGTGCCTTGAACCCCCTCCGCGACTGGGC
10	09-0139C	1.93228E-06	1	5	CALNPLRDWADKLIF	AGTAAGGTCTGAAGACTGTGCCACTTACTACTGTGCCTTGAACCCCCTCCGCGACTGGGC
						* * ******** * ********* **************** ***********

TCRDV03	AGTAAGGACTGAAGACAGTGCCACTTACTACTGTGCCTT
N/D	GAACCCCCTCCGCG
TCRDJ01	ACTGGG

CDR3δ amino acid	number of rearrangements (rank)	number of rearrangements	abundance (rank)	abundance (sum)	present in number of samples	present in number of subjects	number of unique rearrangements	differences compared to germline elements (errors)	nongermline polymorphisms (compared to consensus)	verified unique rearrangements	Reads with errors	total reads	Freq. of reads with errors
CALGEPAPSGIRRSWDTRQMFF	1	16	2	3.79%	8	3	6	9	1	1	99	313,118	0.032%
CALNPLRDWADKLIF	2	15	1	5.42%	6	3	7	9	0	1	48	138,398	0.035%
CAFGGGILYTDKLIF	3	15	65	0.08%	4	3	5	10	0	1	50	2,491	2.007%
CALGEWSVLRLWGTDKLIF	4	13	10	0.52%	7	2	8	7	1	1	24	38,848	0.062%
CALGEASNWGIFDKLIF	7	8	5	1.34%	5	2	4	3	1	1	12	35,783	0.034%
CALGEPFPLLGLYTDKLIF	9	7	4	2.08%	5	2	6	2	0	1	8	54,926	0.015%
CACDTRPAPGGYWTDKLIF	10	7	3	2.62%	6	3	7	1	0	1	5	58,483	0.009%
totals		81			41	18	43	42	3	7	246	642,047	0.04%

B. Analysis of the CALNPLRDWADKLIF (as above), which is the most abundant CDR3δ based on total abundance. 15 rearrangements were detected among three unique subjects (color coded), and in multiple samples per subject. The sequences highlighted in yellow are all identical and are the dominant sequence in subject 09-139, but is also detected in subjects 09-148 and 09-151. The remaining sequences (not highlighted), are all unique and the differences from the consensus sequence are indicated by the red font. Note, that except for a single sequence, all of the differences are in regions of the 3′ V or 5′ J, and hence are likely to be PCR errors since these are regions of the TCR that are germline encoded. This leaves a single sequence that has a sequence difference in the junctional sequence, which possibly represents an independent rearrangement. The germline the 3′ TRDV03 and 5′ TRDJ01 is indicated for references.

C. Summary of seven CDR3δ sequences that were detected in more than one individual. Our analysis found that most "alternative" rearrangements were the result of PCR or sequencing errors. In the end, the contribution of "alternative" rearrangements was small.
